# Virulence and genomic diversity among clinical isolates of ST1 (BI/NAP1/027) *Clostridioides difficile*

**DOI:** 10.1016/j.celrep.2023.112861

**Published:** 2023-07-30

**Authors:** Qiwen Dong, Huaiying Lin, Marie-Maude Allen, Julian R. Garneau, Jonathan K. Sia, Rita C. Smith, Fidel Haro, Tracy McMillen, Rosemary L. Pope, Carolyn Metcalfe, Victoria Burgo, Che Woodson, Nicholas Dylla, Claire Kohout, Anitha Sundararajan, Evan S. Snitkin, Vincent B. Young, Louis-Charles Fortier, Mini Kamboj, Eric G. Pamer

**Affiliations:** 1 Department of Medicine, University of Chicago, Chicago, IL 60637, USA; 2 Duchossois Family Institute, University of Chicago, Chicago, IL 60637, USA; 3 Department of Microbiology and Infectious Diseases, Université de Sherbrooke, Sherbrooke, QC J1E 4K8, Canada; 4 Immunology Program, Memorial Sloan Kettering Cancer Center, New York, NY 10065, USA; 5 Infection Control, Department of Medicine, Memorial Sloan Kettering Cancer Center, New York, NY 10065, USA; 6 Committee on Immunology, University of Chicago, Chicago, IL 60637, USA; 7 Division of Infectious Diseases, Department of Internal Medicine, University of Michigan, Ann Arbor, MI 48109, USA; 8 Department of Microbiology & Immunology, University of Michigan, Ann Arbor, MI 48109, USA; 9 Lead contact

## Abstract

*Clostridioides difficile* produces toxins that damage the colonic epithelium, causing colitis. Variation in disease severity is poorly understood and has been attributed to host factors and virulence differences between *C. difficile* strains. We test 23 epidemic ST1 *C. difficile* clinical isolates for their virulence in mice. All isolates encode a complete Tcd pathogenicity locus and achieve similar colonization densities. However, disease severity varies from lethal to avirulent infections. Genomic analysis of avirulent isolates reveals a 69-bp deletion in the *cdtR* gene, which encodes a response regulator for binary toxin expression. Deleting the 69-bp sequence in virulent R20291 strain renders it avirulent in mice with reduced toxin gene transcription. Our study demonstrates that a natural deletion within *cdtR* attenuates virulence in the epidemic ST1 *C. difficile* isolates without reducing colonization and persistence. Distinguishing strains on the basis of *cdtR* may enhance the specificity of diagnostic tests for *C. difficile* colitis.

## In brief

Dong et al. show that clinical ST1 *C. difficile* isolates with identical pathogenicity loci have variable virulence in mice. A natural deletion within the regulatory gene (*cdtR*) for binary toxin attenuates virulence in epidemic ST1 *C. difficile* isolates without reducing colonization and persistence.

## Graphical Abstract

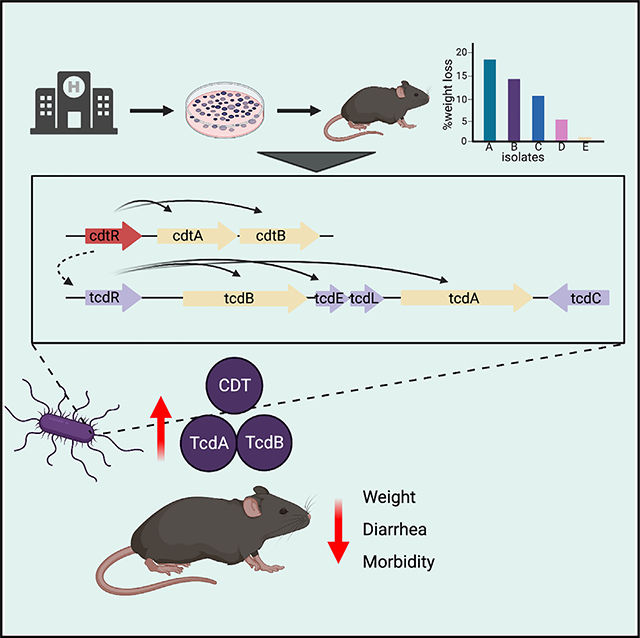

## INTRODUCTION

*Clostridioides difficile* is a gram-positive, spore-forming anaerobic bacterium and the leading cause of nosocomial infections in the United States.^1–3^ Infections are acquired by oral ingestion of *C. difficile* spores, which are prevalent in the environment and can survive for extended periods of time on contaminated surfaces. Upon ingestion, *C. difficile* spores germinate, produce toxins, and cause colitis and, in severe cases, can result in mortality. The major virulence factors of *C. difficile* are toxins A (*tcdA*) and B (*tcdB*), which are encoded in the pathogenicity locus (PaLoc).^4,5^ These toxins glycosylate and thereby inactivate host GTPases, triggering the death of intestinal epithelial cells and leading to gut inflammation.^6^

The *C. difficile* species is comprised of hundreds of strain types across more than 6 phylogenetic clades. PCR- and sequencing-based approaches, including PCR-ribotyping (RT) and multilocus sequencing typing (MLST or ST), have been used to characterize *C. difficile* strain types. Recently, whole-genome sequencing (WGS) has greatly contributed to our understanding of *C. difficile* diversity, evolution, and epidemiology.^7^ Almost two decades ago, the BI/NAP1/027 strain, characterized as ST1 by MLST, emerged as a cause of severe nosocomial outbreaks and increased *C. difficile* infection (CDI) incidence in North America and Europe. Since then, the prevalence of ST1 has declined, but it remains among the most frequently isolated strains in hospital- and community-acquired CDI cases in the United States.^2,8–11^ The ST1 *C. difficile* strain encodes an additional CDT toxin (encoded by *cdtA* and *cdtB* and also referred to as binary toxin), which is an ADP-ribosyltransferase that modifies actin and disrupts cellular cytoskeleton organization.^12^ The ST1 strains have higher minimal inhibitory concentrations (MICs) to several antibiotics, most notably fluroquinolones, and produce higher amounts of TcdA and TcdB compared to non-ST1 strains.^13,14^ The relative virulence of the ST1 strain is controversial, however, with some studies demonstrating clinical disease severities similar to other strains.^15–17^ Host factors can impact the severity of CDI, including underlying diseases, immune competence, and microbiome composition.^18,19^ Whether genetic variants of ST1 explain diverse disease manifestations is unknown.

To determine intra-strain type virulence diversity, we used an antibiotic-treated mouse model of CDI to test a panel of PaLoc- and CdtLoc-encoding ST1 *C. difficile* clinical isolates to quantify disease severity.^20^ Clinical *C. difficile* isolates with identical PaLocs caused a range of disease severities, with two isolates causing no detectable disease in antibiotic-treated wild-type, germ-free mice or MyD88-deficient mice. We identified a 69-bp deletion in the *cdtR* gene of these two avirulent isolates that encodes a LytTR family response regulator that regulates CDT expression. The 69-bp deletion in the *cdtR* leads to reduced CDT toxin and PaLoc gene expression, resulting in loss of virulence and confirming previous studies implicating CdtR as regulator of CDT and Tcd toxin expression.^21^ Overall, our study describes virulence diversity within a single strain type and demonstrates the critical role of CdtR for ST1 *C. difficile* virulence.

## RESULTS

### Clinical ST1 *C. difficile* isolates demonstrate variable severities in mice

**Figure 1.**
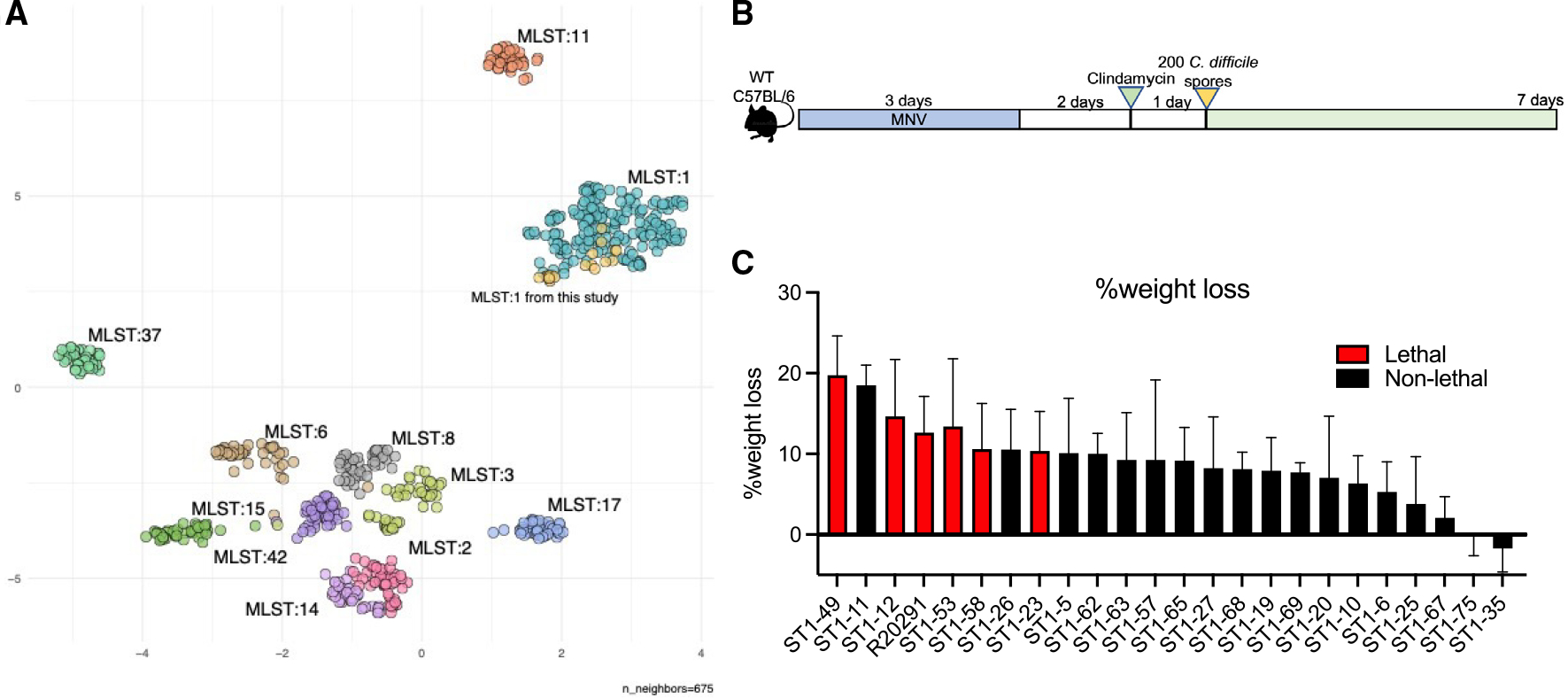
Clinical ST1 *C. difficile* isolates demonstrated variable virulence in mice treated with antibiotics (A) Plot of the UMAP analysis of the presence or absence of unique coding sequences (annotated proteins or unannotated protein clusters) across the top 10 STsof *C. difficile* strains in Patric. (B) Mouse experiment schematic: wild-type C57BL/6 mice were treated with metronidazole, vancomycin, and neomycin (MNV; 0.25 g/L for each) in drinking water for 3 days, followed by one intraperitoneal injection of clindamycin (200 μg/mouse) 2 days after antibiotic recess. Then, mice were inoculated with 200 *C. difficile* spores via oral gavage. Daily body weight and acute disease scores were monitored for 7 days post-infection. (C) Maximum percentage of weight loss to baseline was calculated using the lowest weights within 7 days post-infection divided by day 0 weights. n (number of mice per strain-infected group) = 5–8 except for ST1-62 and ST1-68, which have 2 mice per group. Results represent means ± SD.

We focus on a group of 23 *C. difficile* isolates belonging to ribotype 027 epidemic strains (here referred to as ST1) isolated from patients with diarrhea during a molecular surveillance program at Memorial Sloan Kettering Cancer Center 2013–2017.^22^ Whole-genome Illumina sequencing of these isolates allows us to compare them to public collections. We plotted a uniform manifold approximation and projection (UMAP) analysis of the presence or the absence of unique coding sequences (annotated proteins or unannotated protein clusters) across the top 10 STs of *C. difficile* strains in Patric (date: February 10, 2021).^23^ Different STs cluster individually, and our ST1 isolates overlap with other ST1 *C. difficile* included in the analysis, confirming their strain type (Figure 1A). These 23 isolates demonstrate high genome-wide similarity by average nucleotide identity (ANI) scores above 99.8% and encode identical PaLoc sequences (Figures S1A and S1B). To study if close-related *C. difficile* isolates may have variable virulence, mice treated with antibiotics (metronidazole, neomycin, vancomycin in drinking water with clindamycin intraperitoneal injection) were orally infected with each of these isolates at a dose of 200 spores, and *C. difficile* pathogenicity was monitored throughout a 7-day time course (Figure 1B). Mice infected with different ST1 isolates displayed a spectrum of disease severity, including variable weight loss and mortality (Figures 1C and S1C). The widely used ST1 lab strain R20291 was included in parallel for virulence comparison. Within our ST1 collection, 5 isolates resulted in mortality in mice. A few isolates caused more severe weight loss than R20291, including ST1-49, ST1-11, and ST1-12, while most ST1 isolates caused moderate and non-lethal infections. Two isolates, ST1-75 and ST1-35, demonstrated no impact on mouse body weights. No apparent colonization deficiency was observed in any of these isolates (Figure S1D). The variable pathogenicity induced by a group of ST1 isolates with identical PaLocs suggested additional regulatory mechanisms of *C. difficile* virulence. Therefore, we sought to examine other genomic factors that are responsible for attenuated virulence of *C. difficile* isolates ST1-75 and ST1-35.

### Two ST1 *C. difficile* isolates demonstrate avirulent phenotype

**Figure 2.**
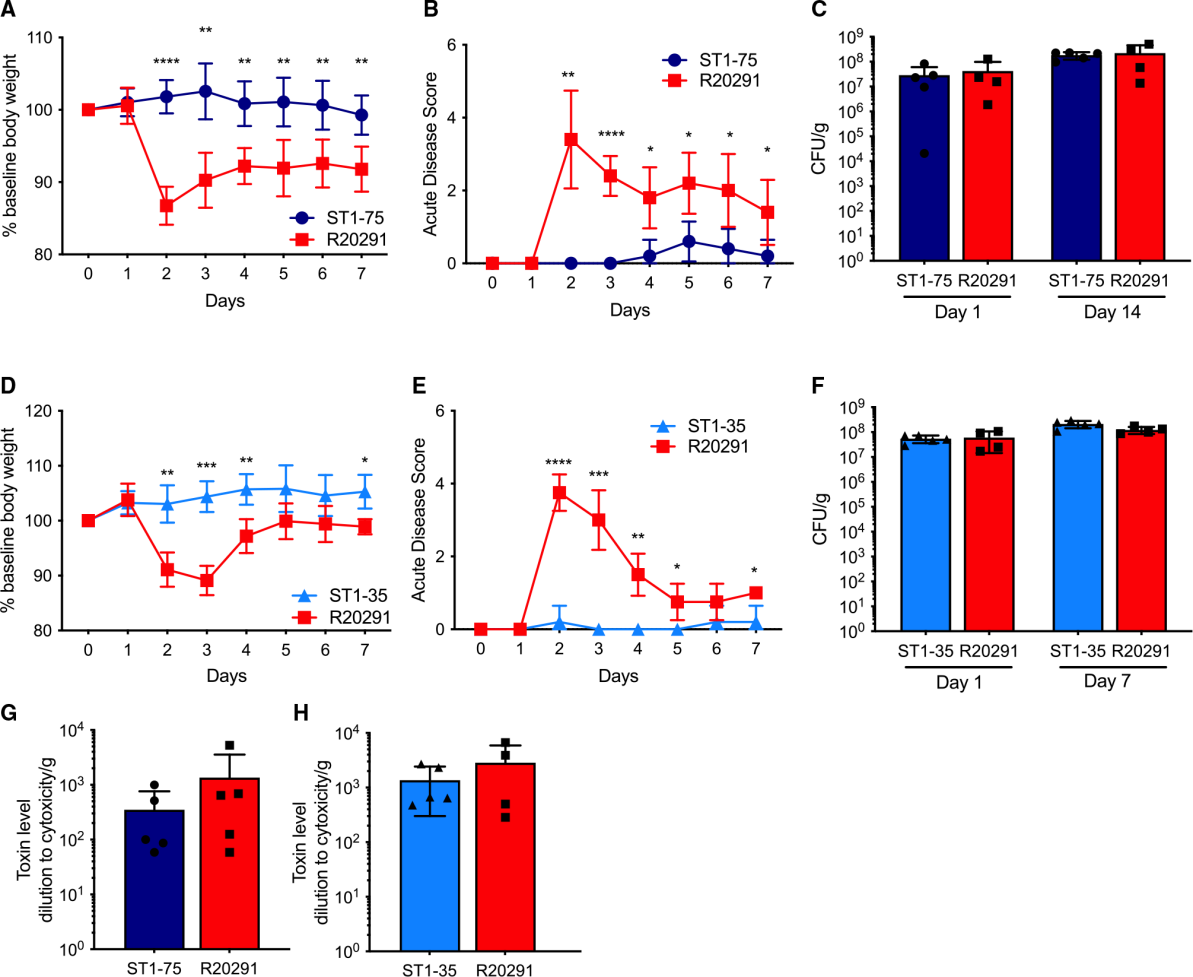
Two isolated clinical strains of *C. difficile* have no virulence in mice treated with antibiotics Wild-type C57BL/6 mice (n = 3–5 per group) were treated with MNV (0.25 g/L for each) in drinking water for 3 days, followed by one intraperitoneal injection of clindamycin (200 μg/mouse) 2 days after antibiotic recess. Then, mice were inoculated with 200 *C. difficile* spores via oral gavage. Daily body weight and acute disease scores were monitored for 7 days post-infection. (A and D) Percentage of weight loss to baseline of mice infected with indicated strains. (B and E) Acute disease scores comprising weight loss, body temperature drop, diarrhea, and morbidity of mice infected with indicated strains. (C and F) Fecal colony-forming units measured by plating on selective agar on indicated days. (G and H) Fecal Tcd toxins measured by CHO cell rounding assay 1 day post-infection. Results represent means ± SD. *p < 0.05, **p < 0.01, ***p < 0.001, ****p < 0.0001.

Among the *C. difficile* isolates that we examined using antibiotic-treated mice, two isolates, ST1-75 and ST1-35, caught our attention due to their strikingly attenuated phenotypes (Figure 1C). Almost no weight loss was observed throughout the 7-day time course, and low acute disease scores were displayed in mice infected with ST1-75 or ST1-35 compared with mice infected with R20291 (Figures 2A, 2B, 2D, and 2E). This avirulent phenotype was not due to colonization deficiency of ST1-75 or ST1-35, as the fecal colony-forming units (CFUs) recovered from the mice infected with these two isolates were comparable to R20291-infected mice on both early and late days post-infection (Figures 2C and 2F). Fecal levels of TcdA and TcdB were also measured, and similar levels were seen in the feces at day +1 post-infection from mice infected with ST1-75 and ST1-35 compared with R20291 (Figures 2G and 2H).

**Figure 3.**
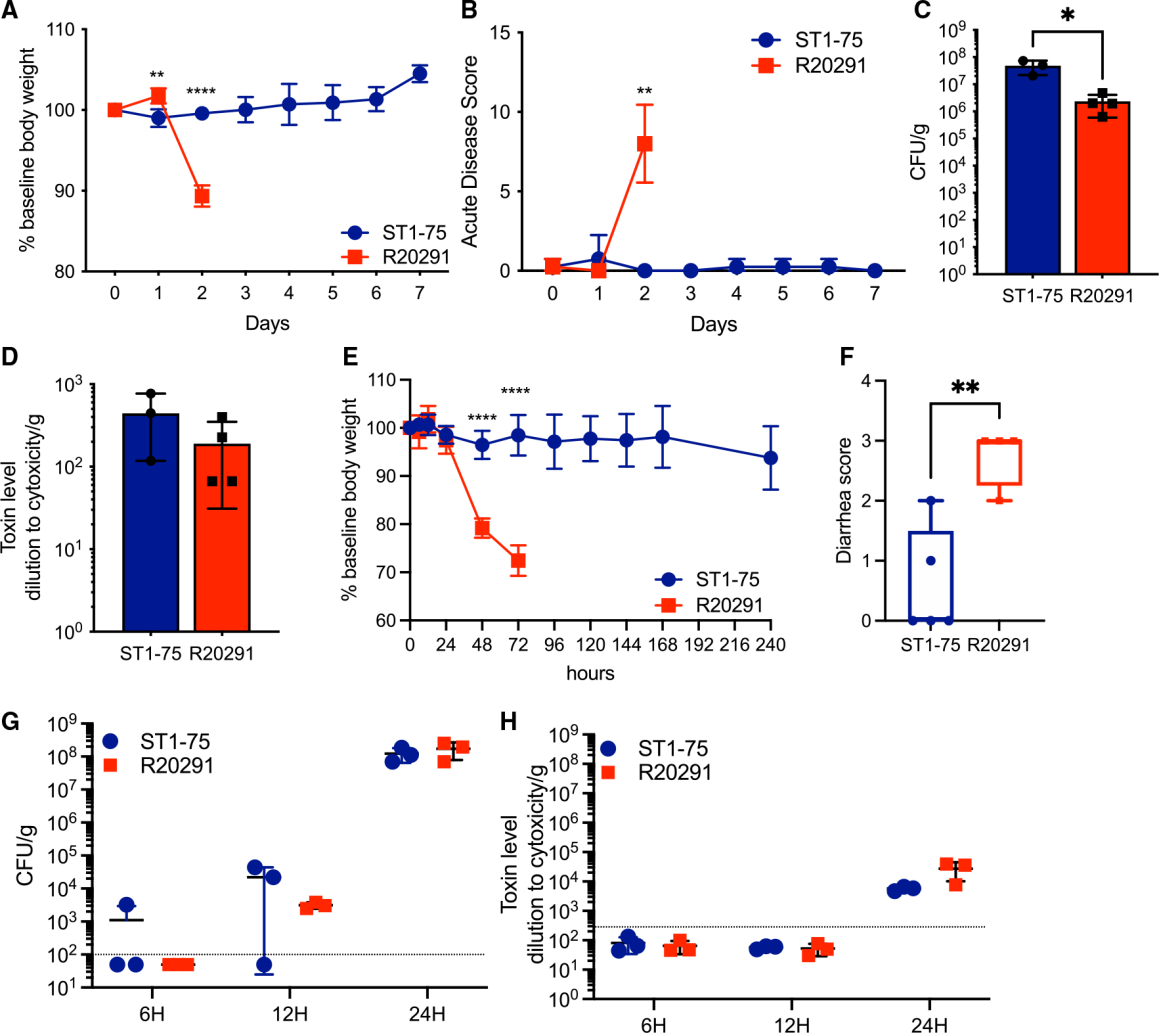
Avirulent *C. difficile* strain demonstrates no virulence in innate immune-deficient mice and germ-free mice (A–D) *MyD88*^−/−^ mice (n = 4 per group) were treated with MNV and clindamycin before being orally administered with 200 spores of *C. difficile* strains. Daily body weight and acute disease scores were monitored for 7 days post-infection. (A) Percentage of weight loss to baseline of mice infected with indicated strains. (B) Acute disease scores comprising weight loss, body temperature drop, diarrhea, and morbidity of mice infected with indicated strains. (C) Fecal colony-forming units measured by plating on selective agar 1 day post-infection. (D) Fecal Tcd toxins measured by CHO cell rounding assay 1 day post-infection. (E–H) Germ-free mice (n = 3 to 5) orally administered with 200 spores of indicated *C. difficile* strains. Daily body weight and acute disease scores were monitored for 10 days post-infection. (E) Percentage of weight loss to baseline of mice infected with indicated strains. (F) Diarrhea scores of mice infected with indicated strains 2 days post-infection. Results represent min to max showing all points. (G) Fecal colony-forming units measured by plating on selective agar at 6, 12, and 24 h post-infection. (H) Fecal Tcd toxins measured by CHO cell rounding assay at 6, 12, and 24 h post-infection. Results represent means ± SD. *p < 0.05, **p < 0.01, ****p < 0.0001.

To further investigate this avirulent phenotype, we inoculated ST1-75 into *MyD88*^−/−^ mice, which lack the adaptor protein for Toll-like receptor signaling.^24^ *MyD88*^−/−^ mice fail to recruit neutrophils to the colonic tissue during early stages of CDI and display markedly increased susceptibility to *C. difficile*-induced colitis.^25^ Here, *MyD88*^−/−^ mice were treated with antibiotics and infected with either ST1-75 or R20291. Mice infected with R20291 quickly succumbed to infection 2 days after spore inoculation, whereas all *MyD88*^−/−^ mice infected with ST1-75 survived the experiment with minimal weight loss or disease scores (Figures 3A and 3B). Consistent with our results with wild-type mice, no deficiencies of colonization or Tcd toxin production were observed day +1 post-infection of *MyD88*^−/−^ mice (Figures 3C and 3D). These data suggest that the attenuation of the avirulent strain is independent of MyD88-mediated host innate immunity.

Germ-free mice are highly susceptible to CDI because they lack microbiome-mediated colonization resistance against *C. difficile*.^26,27^ To investigate whether the gut microbiome renders ST1-75 avirulent, germ-free mice were infected with ST1-75 or R20291. Similarly, we observed no mortality or weight loss in the mice with ST1-75 infection, whereas mice infected with R20291 quickly lost weight and died (or had >20% weight loss) (Figure 3E). Milder diarrhea was observed in mice with ST1-75 compared with R20291 (Figure 3F). We observed no differences in colonization or fecal Tcd toxins between ST1-75 and R20291 up to 24 h post-infection (Figures 3G and 3H). Similar attenuation was also seen in ST1-35-infected germ-free mice (Figure S2). In contrast, isolates that demonstrated relatively mild pathogenicity in antibiotic-treated mice, such as ST1-25 and ST1-67 (Figure 1C), led to severe weight loss and diarrhea in germ-free mice (Figure S2), reaffirming the protective role of the gut microbiome during CDI. However, the attenuation of ST1-75 and ST1-35 in mice is independent of the gut microbiome.

### Prophages identified in avirulent strains do not impact ST1 *C. difficile* virulence

We next sought to determine the genetic factors that may abrogate *C. difficile* virulence in ST1-75 and ST1-35. Fully circularized genomes of 14 ST1 isolates were successfully obtained using Nanopore and Illumina hybrid assembly, and pangenomic analysis was conducted on these 14 genomes and R20291 using the anvi’o pangenomics workflow.^28^ A group of gene clusters that are unique to ST1-75 and ST1-35, which are enriched for phage-related genes, stood out (Figure S3A). We then applied PHASTER, a tool for phage identification in bacterial genomes, to discover two prophages in the genomes of ST1-75 and ST1-35. One prophage resides on a 41-kb plasmid in ST1-75 and ST1-35 with 4–5 copies per cell and here is named phiCD75-2. Blasting phiCD75-2 found high similarities to reported *C. difficile* phages phiCD38-2 (99.8% identity) and phiCDHS1 (94.7% identity).^29–32^ In addition, a 54-kb segment was found as inserted into the chromosomal DNA of ST1-75 and ST1-35 around position 1.29 Mbp and here is named phiCD75-3. phiCD75-3 does not show high similarity to any described *C. difficile* phages to date.

Lysogenic bacteriophages have been identified in many *C. difficile* genomes and play an important role in shaping *C. difficile* evolution. However, their roles in *C. difficile* biology, especially virulence, are not well characterized.^33,34^ To investigate the potential role of these two prophages on C. *difficile* virulence, we induced lytic phage particles of phiCD75-2 and phiCD75-3 from ST1-75 culture and infected R20291 to generate R20291 lysogens harboring these prophages. We were able to generate R20291 derivatives carrying phiCD75-2, phiCD75-3, or both prophages in their genomes (Figure S3B). WGS of lysogenic R20291 strains confirmed that phiCD75-3 was inserted *in situ* as in ST1-75 at 1.29 Mbp. Antibiotic-treated mice infected with R20291 lysogenic strains (Figure S3B) followed the curve of virulent infection, as 10% weight loss and 4–6 disease scores were seen during the peak of symptomatic infection (Figures S3C and S4D). The seemingly faster recovery in the lysogens was not a reproducible finding. Similar levels of colonization and Tcd toxin production were also observed (Figures S3E–S3H). Here, we discover two prophages in avirulent strains ST1-75 and ST1-35 that are not present in R20291 or other ST1 strains from our collection. However, these two prophages do not appear to impact the virulence of R20291 in antibiotic-treated mice.

### Mutations in the *cdtR* gene eliminate ST1 *C. difficile* virulence in mice

**Figure 4.**
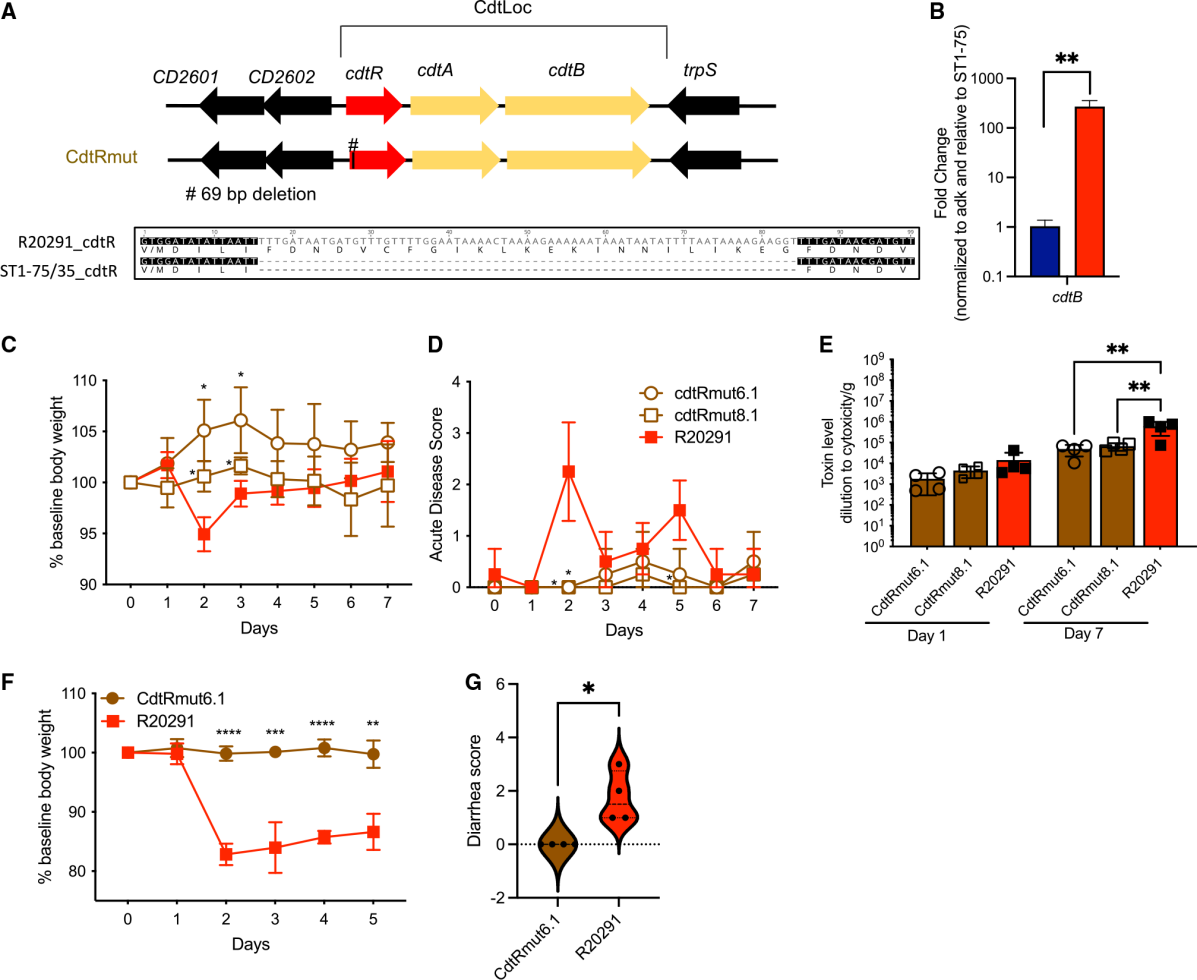
Binary toxin regulator *cdtR* contributes to *C. difficile* virulence in mice (A) Deletion identified in ST1-35/75 and schematic of *cdtR* mutants generated using R20291 *C. difficile* strain. (B) Germ-free mice (n = 3 per group) orally administered with 200 spores of indicated *C. difficile* strains. Binary toxin gene *cdtB* transcripts were measured by RT-qPCR on cecal contents harvested at 24 h post-infection with 2 technical replicates per sample. Transcripts were normalized to the adk, and fold change is relative to ST1-75 condition. (C–E) Wild-type C57BL/6 mice (n = 3 to 5 per group) were treated with MNV and clindamycin as previously described. Then, mice were inoculated with 200 *C. difficile* spores via oral gavage. Daily body weight and acute disease scores were monitored for 7 days post-infection. (C) Percentage of weight loss to baseline of mice infected with indicated strains. Both CdtRmut6.1 and CdtRmut8.1 have significant differences on days 2 and 3 compared with R20291. (D) Acute disease scores comprising weight loss, body temperature drop, diarrhea, and morbidity of mice infected with indicated strains. CdtRmut6.1 has a significant difference on day 2 compared with R20291. CdtRmut8.1 has a significant difference on days 2 and 5 compared with R20291. (E) Fecal Tcd toxins measured by CHO cell rounding assay on indicated days. (F and G) Germ-free mice (n = 4) orally administered with 200 spores of indicated *C. difficile* strains. Daily body weight and were monitored for 5 days post-infection. (F) Percentage of weight loss to baseline of mice infected with indicated strains. (G) Diarrhea scores of mice infected with indicated strains 3 days post-infection (violin plot showing all points). Results represent means ± SD. *p < 0.05, **p < 0.01, ***p < 0.001, ****p < 0.0001.

Lysogenic R20291 strains with either or both prophages did not recapitulate the avirulent phenotype of ST1-75 or ST1-35. A closer look at the chromosomal genomes of ST1-75 and ST1-35 led us discover a common mutation in their *cdtR* gene, which was reported previously as a transcriptional regulator for binary toxin (CDT) genes, *cdtA* and *cdtB*.^35^ A 69-bp deletion was found in the *cdtR* gene of ST1-75 and ST1-35, leading to an in-frame deletion of 23 amino acids (Figure 4A). To investigate if there is a possible loss of function of CdtR resulting from the deletion, we accessed the transcriptional level of *cdtB* in mouse cecum following infection of ST1-75 or R20291. More than a 2-log reduction of *cdtB* transcripts was observed in the ST1-75 group (Figure 4B), suggesting an important role of these 69 bp for a fully functional *cdtR* gene. Next, we applied a CRISPR-mediated genome editing approach to generate CdtR mutants using the parental R20291 strain to study the contribution of CdtR to *C. difficile* virulence (Figures S4A and 4A). In accordance with a previous report,^21^ knocking out *cdtR* either by deleting the whole gene (CdtRKO8.1 and CdtRKO10.3) or by introducing a proximal premature stop codon (CdtRstop4.2 and CdtRstop8) led to a loss of pathogenicity in antibiotic-treated mice (Figures S4B and S4C), confirming a critical role of CdtR for *C. difficile* virulence. Moreover, deleting the exact same 69-bp region, as in ST1-75/35, in the *cdtR* of R20291 (CdtRmut6.1 and CdtRmut8.1) again eliminated the virulence of *C. difficile* (Figures 4C and 4D). Thus, loss of the 69 bp in *cdtR* explains the avirulence phenotype of ST1-75/35. On the other hand, colonization of these CdtR mutants, assessed by CFUs at day+1 post-infection, was comparable to that of R20291 (Figures S4D and S4G), suggesting that CdtR is not required for colonization. Interestingly, while the fecal levels of Tcd toxins of CdtR mutants were comparable to R20291 in the early phase (day+1 post-infection), a significantly reduced level at a later time point (7 days post-infection) was observed upon infection of CdtR mutants (Figures S4E, S4F, 4E and S4H), supporting a role of CdtR in regulating Tcd toxin production. Further, infecting germ-free mice with CdtRmut6.1 results in no weight loss or diarrhea, perfectly recapitulating ST1-75 and ST1-35 phenotypes in germ-free mice (Figures 4F, 4G, and S4I). Collectively, CRISPR-edited CdtR mutant strains mimic phenotypes of ST1-75 and ST1-35 in mice, demonstrating that the *cdtR* gene is necessary for *in vivo* virulence. Furthermore, the 69-bp region in *cdtR*, which is deleted in ST1-75/35, is necessary for proper CdtR function through mechanisms yet to be determined.

### Mutation in *cdtR* reduce Tcd toxin transcription *in vivo*

**Figure 5.**
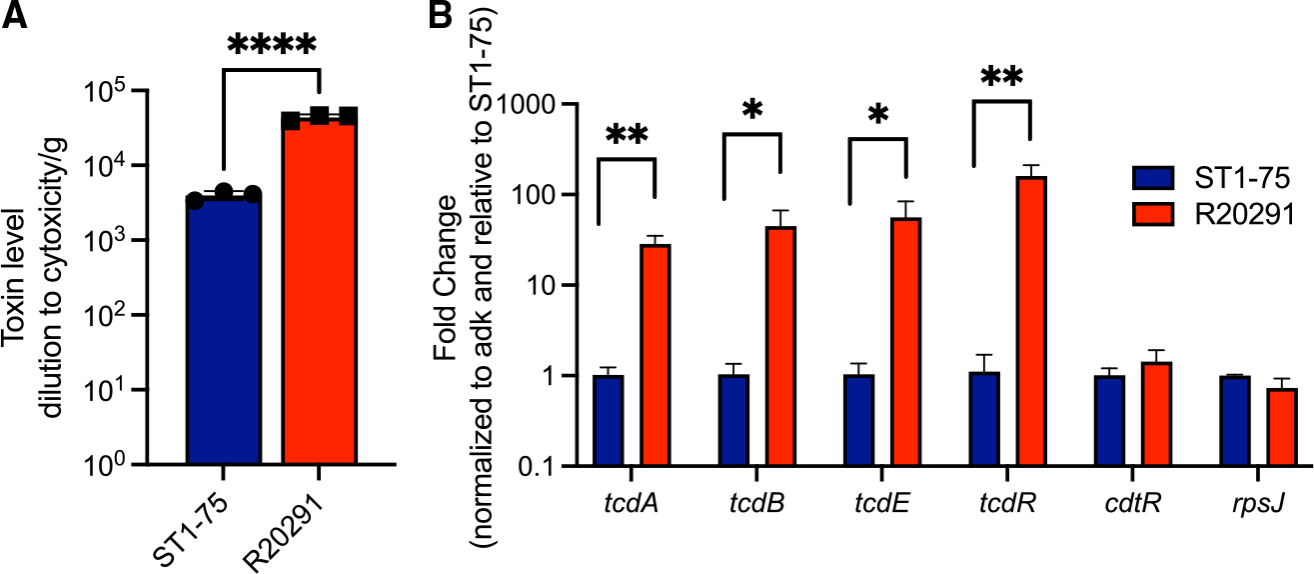
CdtR regulates Tcd toxins transcription *in vivo* Germ-free mice (n = 4) were orally administered with 200 spores of indicated *C. difficile* strains and cecal contents were harvested at 24 h post-infection. (A) Cecal Tcd toxins measured by CHO cell rounding assay. (B) Indicated gene transcripts were measured by RT-qPCR with 2 technical replicates per sample. Transcripts were all normalized to the *adk*, and fold change is relative to ST1-75 for each of the genes. Results represent means ± SD. *p < 0.05, **p < 0.01, ****p < 0.0001.

CdtR mutants produce significantly reduced fecal Tcd toxins at a later time point (7 days post-infection) (Figures 4E and S4E), a phenotype that was confirmed in ST1-75 and ST1-35 (Figures S5A and S5B). To examine whether the 69-bp deletion in *cdtR* impacts Tcd toxin production, we harvested cecal contents from germ-free mice infected with ST1-75 or R20291. In contrast to fecal toxin concentrations, we observed a significantly reduced cecal Tcd toxin concentration in mice infected with ST1-75 compared with R20291 (Figure 5A). This was not due to a slightly lower CFU of cecal ST1-75 in germ-free mice (Figures S5C and S5D). We further validated the reduced toxin production in cecal content by RT-qPCR, and we observed a 50-fold reduction of the tcdA and tcdB transcripts in the cecum of mice infected with ST1-75 (Figure 5B). Additionally, transcripts of other PaLoc genes including *tcdE* and *tcdR* were also reduced in the cecum of mice infected with ST1-75 (Figure 5B). *TcdE* is a putative holin that mediates toxin secretion.^36–38^ Reduced *tcdE* likely further impacts the amount of toxins that may reach the intestinal epithelium. TcdR is a positive regulator of the PaLoc^39,40^ and is likely the common target of CdtR, which results in the observed downregulation of many PaLoc genes. Interestingly, *cdtR* transcripts were comparable between ST1-75 and R20291, suggesting that the 69-bp deletion does not impact the transcript’s stability. These results were further confirmed with the CdtRmut6.1 strain, though to a lesser extent (Figure S5E). Overexpressing wild-type CdtR (but not CdtR with a 69-bp deletion) restored and enhanced the transcription of both PaLoc genes and binary toxin (Figures S5F and S5G). Collectively, we demonstrate that a natural mutation found in *cdtR* of two ST1 clinical isolates results in reduced binary toxin production, reduced Tcd toxin production (and likely secretion) in cecum of infected mice, and attenuated *C. difficile* virulence, independent of host innate immunity, colonization burden, microbiome constitution, or any noticeable impact of incidentally discovered prophages within these strains. This difference of toxin production in cecum at 24 h post-infection is, however, not reflected in feces in parallel but could be reflected at later time points, likely due to cumulative differences over time.

### *cdtR* is versatile and more prevalent than *cdtA* and *cdtB*

**Figure 6.**
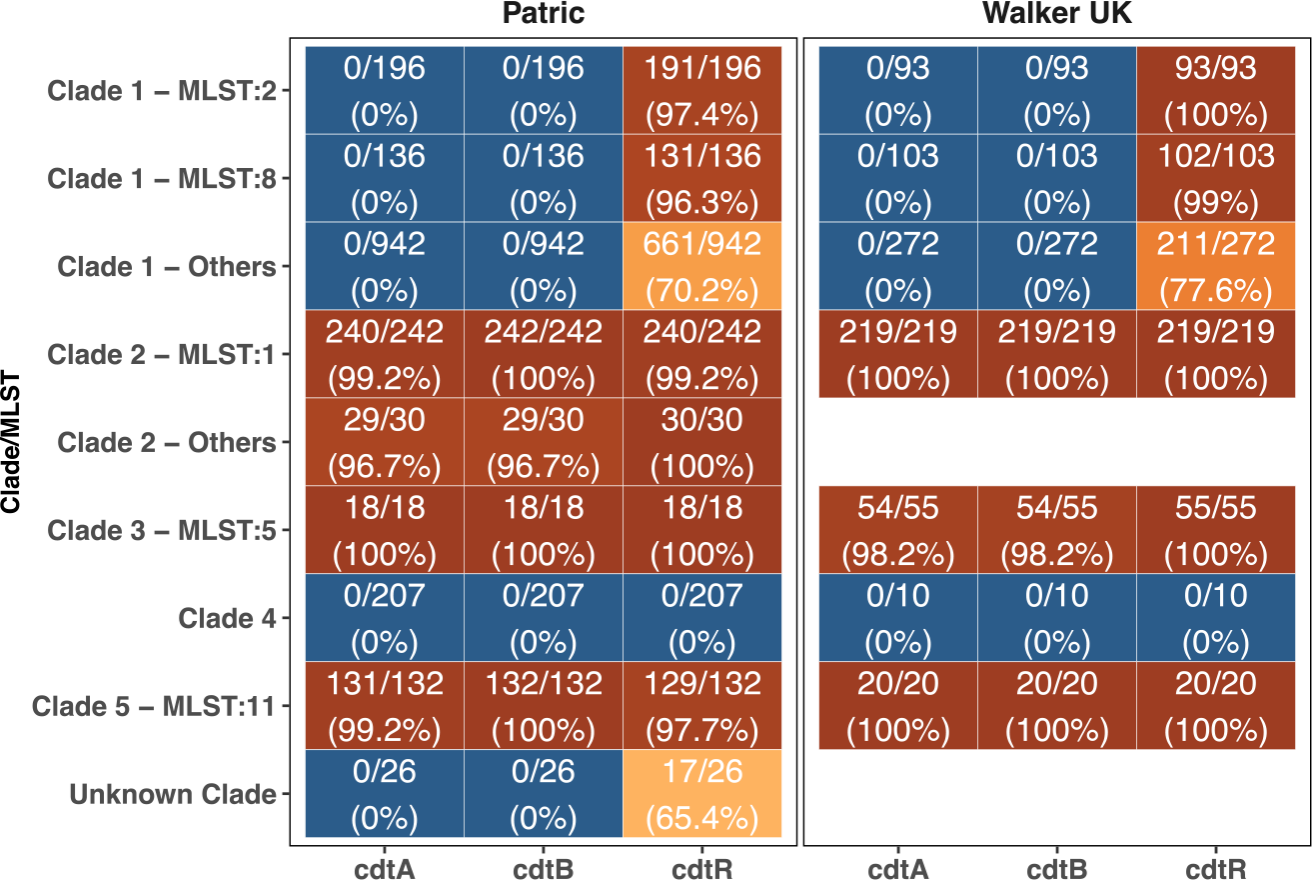
Binary toxin regulator *cdtR* is prevalent in clinical *C. difficile* isolates Binary toxins *cdtA*, *cdtB*, and *cdtR* from R20291 were used as query to BLAST against the assembled contigs. Hits with at least 85% identity and 85% coverage of the query were considered a valid match. Numbers of match in total and percentages are presented.

Our data support a regulatory role of CdtR outside CdtLoc, so we hypothesize that CdtR may have evolved to impact virulence beyond regulating CDT binary toxins. To test this possibility, we surveyed the presence of *cdtR*, *cdtA*, and *cdtB* in two major C. *difficile* clinical collections.^23,41^ As expected, the majority of clade 2 strains, including the epidemic ST1/RT027 strains, contain the CdtLoc with the presence of all three genes. Other subgroups of *C. difficile* strains, including MLST5 and MLST11, were also reported to encode CDT (Figure 6).^42,43^ Unexpectedly, many strain types of *C. difficile* that were reported as CDT negative also encode *cdtR*, such as MLST2 and MLST8 from clade 1 (Figure 6). The higher prevalence of *cdtR* than *cdtA* and *cdtB* supports the possibility that CdtR functions beyond regulating CDT. Additional work is needed to evaluate the functions of CdtR in these CDT-negative strains. Alternatively, CdtR may also lose its function in CDT-positive strains. Two such cases are ST11 strains, in which *cdtR* has lost a premature stop codon resulting in a pseudogene,^42^ as well as here with the mutation in the *cdtR* of ST1-75/35. To evaluate the prevalence of *cdtR* mutations that may lead to a loss of function, we aligned all *cdtR* genes in MLST1 strains from the two described collections and found that several strains had similar truncations at the proximal end that may have lost CdtR function, yet we did not find, within almost 500 strains, the exact deletion we identified in ST1-75/35 (Figure S6A).

The high genetic and phenotypic similarity between ST1-75 and ST-35 led us to investigate their potential clonality. We performed a core-genome SNP analysis across all ST1 isolates from our collection with R20291 as the reference. ST1-75 and ST1-35 shared all SNPs when compared to the genome of R20291 (Figure S6B). Additional clinical evidence supporting that ST1-35 and ST1-75 are clonal is that these strains were isolated from two patients who were hospitalized in the same hospital room within 2 weeks of each other (Figure S6C).

## DISCUSSION

Mouse models are valuable tools to study how *C. difficile* strain variations may result in variable disease severities, thanks to the advantages of their identical genetic, immune background and controlled microbiome compositions. Here, we focused on a group of clinical *C. difficile* isolates belonging to the RT027/MLST1, with high genomic similarity, that all encode PaLoc and CdtLoc. We found that these similar *C. difficile* isolates caused variable disease severities in mice and that a very specific mutation in the *cdtR* gene rendered two clinical isolates, ST1-75 and ST1-35, avirulent. Avirulence was solely dependent on the *cdtR* mutation, as we obtained similar observations using *MyD88*^−/−^ mice and germ-free mice, which was also further validated with CRISPR-edited *cdtR* mutants. Lower transcripts of binary toxin gene *cdtB*, toxin A *tcdA*, and toxin B *tcdB*, together with other PaLoc genes including regulator *tcdR* and putative holin *tcdE*, were observed in germ-free mouse cecum infected with the CdtR mutants. Our data support a critical role of CdtR in regulating *C. difficile* toxin production and secretion, which are essential to ST1 virulence. However, all the other ST1 isolates in this study encoded an intact CdtLoc with wild-type *cdtR*, whose variations in virulence are likely attributable to alternative mechanisms.

The presence of a binary toxin locus has been associated with epidemic strains and hypervirulence of *C. difficile*.^44,45^ CDT belongs to the family of ADP-ribosylating toxins that consist of two components: CDTa (*cdtA*), the enzymatic active ADP-ribosyltransferase that modifies cellular actin, and CDTb (*cdtB*), the binding component that facilitates CDTa translocation. However, despite knowing their enzymatic activities, experimental evidence is very limited to support critical roles of CDT in *C. difficile* virulence.^46^ CDTb was reported to induce Toll-like receptor 2 (TLR2)-dependent pathogenic inflammation, which suppresses a protective eosinophilic response and enhances virulence of RT027 strains; however, *C. difficile* lacking CDTb still causes acute disease in mice.^47^ On the other hand, CdtR, as the transcriptional regulator of *cdtA* and *cdtB*,^6,35,48^ has been previously linked to Tcd toxin production,^21^ suggesting a role as a major virulence regulator. Here, we demonstrated a critical role of CdtR as a determinant of *C. difficile* virulence within ST1 strains. A natural 69-bp deletion in *cdtR* that was found in two clinical isolates can reverse the virulence of a wild-type strain by downregulating the expression of PaLoc genes and binary toxin genes. Additionally, higher prevalence of *cdtR* over *cdtA* or *cdtB* was found while surveying CdtLoc on clinical isolates from public databases. This suggests that CdtR may have evolved to function beyond regulating *cdtA* and *cdtB*. Systematically examining the target genes of CdtR may give us insights on its additional functions, which may also help unveil the mechanisms by which CdtR regulates the PaLoc genes.

ST1-75 and ST1-35 are avirulent in susceptible mouse models despite producing toxins, albeit at reduced levels. This is intriguing because it is well appreciated that toxin expression is necessary for *C. difficile* virulence.^46,49^ However, our data indicate that toxin production is not sufficient for causing CDI. The amount of toxin being produced and released likely impacts the development of disease. The patients from whom we isolated ST1-75 or ST1-35 had an overall mild clinical assessment, and their symptoms may be attributable to causes other than CDI. Current CDI diagnoses largely depend on the detection of the *TcdB* gene or toxin B positivity in feces and may lead to overdiagnosis of CDI. We, together with other reports, suggest the importance of quantifying toxins to evaluate CDI cases.^50–52^ Incorporating adjunctive biomarkers, such as interleukin-1β (IL-1β), better distinguishes CDI from asymptomatic carriage and non-CDI diarrhea.^53^ Here, CdtR regulates both toxin production and secretion and is essential for *C. difficile* virulence in mice, suggesting that it may serve as an adjunctive biomarker for CDI diagnosis.

Apart from characterizing CdtR, we also identified two prophages in ST1-75 and ST1-35. Prophages have been identified in many *C. difficile* genomes and play important roles in shaping *C. difficile* evolution.^33^ While prophages are highly prevalent in *C. difficile*, little is known about how prophages impact *C. difficile* biology. A couple of pioneering studies have shown that prophages can affect *C. difficile* gene expression, impacting toxin production.^29,54,55^ In this study, we identified two prophages in ST1-75/35 and named them phiCD75-2 and phiCD75-3. By making R20291 lysogenic strains harboring either or both prophages, we observed minimal impacts on *C. difficile* virulence by both of the prophages in antibiotic-treated mice. PhiCD38-2 was shown to increase PaLoc gene expression and toxin production in some RT027 isolates but not in all of them, suggesting that the genetic background influences the impact of a newly acquired prophage.^29^ This may explain why phiCD75-2 (a phiCD38-2 derivative) did not increase toxin production in ST1-75 isolates. Certain phages also impact phase variation of the cell surface protein and biofilm formation and carry genes involved in quorum sensing, inferring their roles in bacterial fitness.^30,56,57^ It would be very intriguing to investigate how phiCD75-2 and phiCD75-3 may impact C. *difficile* fitness, including gene expression, antibiotic resistance, and interspecies competition.

In summary, we demonstrate that ST1 *C. difficile* clinical isolates with identical PaLoc display variable virulence *in vivo*. Among them, two clonal clinical isolates, ST1-75 and ST1-35, were avirulent in mice due to a 69-bp deletion mutation in their *cdtR* genes. These data suggest that specific *cdtR* genetic variants within the same strain type may predict disease occurrence and severity. Routine detection of these variants may enhance the specificity of nucleic acid amplification tests (NAATs) for CDI diagnosis. Our data also corroborate recent clinical observations that toxin detection is unreliable as the sole criterion to distinguish between CDI and colonization.

### Limitations of the study

It is acknowledged that the mouse model of CDI does not mimic all aspects of the human infection. The wide range of disease severities we observed in mice does not perfectly mirror the clinical data. Thus, phenotypes in mice cannot be used to infer CDI severity in humans. However, the identical background of mice led us to discover essential factors for *C. difficile* virulence that were masked by the complex host factors within clinical data. We discovered an important role of CdtR in regulating Tcd and CDT toxins in clinical ST1, a prevalent *C. difficile* strain type. This critical role of CdtR for virulence may not be generalizable to all other strain types. For example, ST11 has a CdtR pseudogene, and ST37 has no CDT genes, while both still cause CDI in patients. Better understanding the roles of CdtR in other strain types will further help dissect the contributions of Tcd and CDT toxins in clinical CDI.

## STAR★METHODS

Detailed methods are provided in the online version of this paper and include the following:

### RESOURCE AVAILABILITY

#### Lead contact

Further information and requests for resources and reagents should be directed to and will be fulfilled by the lead contact, Qiwen Dong (qiwendong0721@gmail.com)

#### Materials availability

All unique reagents and plasmids generated in this study are available from the lead contact Qiwen Dong (qiwendong0721@gmail.com) with a completed materials transfer agreement.

#### Data and code availability

- Whole-genome sequence data were uploaded to National Center for Biotechnology Information (NCBI) Sequence Read Archive (SRA) under BioProject: PRJNA885086 and PRJNA595724.
- This paper does not report original code.
- Any additional information required to reanalyze the data reported in this work paper is available from the lead contact upon request.

### EXPERIMENTAL MODEL AND STUDY PARTICIPANT DETAILS

#### Bacterial strains and growth conditions

*C. difficile* isolates were grown on brain heart infusion (BHI) agar plates supplemented with yeast extract and L-cysteine (BHIS) or in BHIS broth at 37°C in an anaerobic chamber (Coylabs). Antibiotics may be supplemented as described in the detailed methods. *B. subtilis* and *E. coli* were routinely grown on Luria-Bertani (LB) agar or in LB broth at 37°C aerobically. Transformed *B. subtilis* and *E. coli* with plasmids were selected with media containing 15 μg/mL chloramphenicol or 100 μg/mL ampicillin as needed.

#### Cell line

Chinese hamster ovary cells (CHO/dhFr-, ATCC#CRL-9096) were grown in complete media (alpha-modified MEM supplemented with 10% FBS, 2% HEPES, 0.4% L-Glutamine, 0.007% β-mercaptoethanol, 1% Penicilin/Streptimycin/Gentamycin) at 37°C with 5% CO2 and split every 2–3 days for subculture.

#### Mice

Wild-type female C57BL/6 mice, aged 6 to 8 weeks, were purchased from the Jackson Laboratories. Male and female *MyD88*^−/−^ mice were maintained in augmentin (0.48 g/L and 0.07 mg/L of amoxicillin and clavulanate respectively) in the drinking water in specific-pathogen-free (SPF) facility at the University of Chicago. Male and female germ-free C57Bl/6J mice were bred and maintained in plastic gnotobiotic isolators within the University of Chicago Gnotobiotic Core Facility and fed ad *libitum* autoclaved standard chow diet (LabDiets 5K67) before transferring to BSL2 room for infection. Mice housed in the BSL2 animal room are fed irradiated feed and provided with acidified water. All mouse experiments were performed in compliance with University of Chicago’s institutional guidelines and were approved by its Institutional Animal Care and Use Committee.

### METHOD DETAILS

#### *C. difficile* clinical isolate collection and classification

Toxigenic *C. difficile*-positive stool specimens were collected at Memorial Sloan Kettering Cancer Center between 2013 and 2017. *C. difficile* isolates were recovered by plating onto BHIS agar, supplemented with antibiotics D-cycloserine and cefoxitin (BHI and yeast extract were from BD Biosciences, and the other components were from Sigma-Aldrich) in an anaerobic chamber (Coylabs). Individual colonies that were able to grow in the presence of these antibiotics and that had the characteristic phenotype of *C. difficile* were selected, isolated, and subjected to whole-genome sequencing and MLST classification.^75^

#### *C. difficile* spore preparation and numeration

*C. difficile* sporulation and preparation was processed as described previously^76^ with minor modifications. Briefly, single colonies of *C. difficile* isolates were inoculated in deoxygenated BHIS broth and incubated anaerobically for 40–50 days. *C. difficile* cells were harvested by centrifugation and five washes with ice-cold water. The cells were then suspended in 20% (w/v) HistoDenz (Sigma, St. Louis, MO) and layered onto a 50% (w/v) HistoDenz solution before centrifugating at 15,000 × g for 15 min to separate spores from vegetative cells. The purified spores pelleted at the bottom were then collected and washed for four times with ice-cold water to remove traces of HistoDenz, and finally resuspended in sterile water. Prepared spores were heated to 60°C for 20 min to kill vegetative cells, diluted and plated on both BHIS agar and BHIS agar containing 0.1% (w/v) taurocholic acid (BHIS-TA) for numeration. Spore stocks for mouse infection were verified to have less than 1 vegetative cell per 200 spores (as the infection dose).

#### Virulence assessment of clinical isolates in mice

SPF mice were treated with antibiotic cocktail containing metronidazole, neomycin and vancomycin (MNV) in drinking water (0.25 g/L for each antibiotic) for 3 days, 2 days after removing MNV, the mice were received one dose of clindamycin (200 μg/mouse) via intraperitoneal injection. Mice were then the next day infected with 200 *C difficile* spores via oral gavage. Germ-free mice were infected with 200 *C difficile* spores via oral gavage without antibiotic treatments.

Following infection, mice were monitored and scored for disease severity by four parameters^77^: weight loss (>95% of initial weight = 0, 95%–90% initial weight = 1, 90%–80% initial weight = 2, <80% = 3), surface body temperature (>95% of initial temp = 0, 95%–90% initial temp = 1, 90%–85% initial temp = 2, <85% = 3), diarrhea severity (formed pellets = 0, loose pellets = 1, liquid discharge = 2, no pellets/caked to fur = 3), morbidity (score of 1 for each symptoms with max score of 3; ruffled fur, hunched back, lethargy, ocular discharge).

#### Quantification of fecal colony forming units

Fecal pellets or cecal content from *C. difficile* infected mice were harvested and resuspended in deoxygenated phosphate-buffed saline (PBS), diluted and plated on BHI agar supplemented with yeast extract, taurocholic acid, L-cysteine, D-cycloserine and cefoxitin (CC-BHIS-TA) at 37°C anaerobically for overnight.^78^

#### Cell-based assay to quantify fecal and cecal Tcd toxin

The presence of *C. difficile* Tcd toxins was determined using a cell-based cytotoxicity assay as previously described with minor modifications.^78^ Briefly, Chinese hamster ovary cells (CHO/dhFr-, ATCC#CRL-9096) were incubated in a 96-well plate overnight at 37°C. 10-fold dilutions of supernatant from resuspended fecal or cecal content were added to CHO/dhFr-cells, incubated overnight at 37°C. Cell rounding and death was scored the next day. The presence of *C. difficile* Tcd toxins was confirmed by neutralization by antitoxin antisera (Techlab, Blacksburg, VA). The data are expressed as the log10 reciprocal value of the last dilution where cell rounding was observed.

#### DNA extraction, RNA extraction and reverse transcription

Fecal DNA was extracted using DNeasy PowerSoil Pro Kit (Qiagen), and RNA was isolated from cecal contents or bacterial culture using RNeasy PowerMicrobiome Kit (Qiagen) according to the manufacturer’s instructions, respectively. Complementary DNA was generated using the QuantiTect reverse transcriptase kit (Qiagen) according to the manufacturer’s instructions.

#### Quantitative polymerase chain reaction (qPCR)

Quantitative PCR was performed on genomic DNA or complementary DNA using primers (listed in Table S1) with PowerTrack SYBR Green Master Mix (Thermo Fisher). Reactions were run on a QuantStudio 6 pro (Thermo Fisher). Relative abundance was normalized by ΔΔCt.

#### Generation of *C. difficile cdtR* mutants using CRISPR

CRISPR editing on *C. difficile* strains R20291 was performed as described in.^61^ The primers were listed in Table S1.^79–83^ Briefly, donor regions for homology were generated by separately amplifying regions ~500 bp upstream and ~500 bp downstream of the target of interest. The resulting regions were cloned into pCE677 between NotI and XhoI sites by Gibson Assembly. Geneious Prime (v11) was used to design sgRNAs targeting each deleted target. sgRNA fragments were then amplified by PCR from pCE677, using an upstream primer that introduces the altered guide and inserted at the MscI and MluI sites of the pCE677-derivative with the appropriate homology region. Regions of plasmids constructed using PCR were verified by Sanger sequencing. Plasmids were then passaged through NEBturbo *E. coli* strain before transformation into *Bacillus subtilis* strain BS49. The CRISPR-Cas9 deletion plasmids which harbor the oriT (Tn916) origin of transfer, were then introduced into *C. difficile* strains by conjugation.^84^ *C. difficile* colonies were then screened for proper mutations in the genomes by PCR and correct clones were further validated by whole-genome sequencing.

#### Whole-genome sequencing and assembly

DNA was extracted using the QIAamp PowerFecal Pro DNA kit (Qiagen). Libraries were prepared using 100 ng of genomic DNA using the QIAseq FX DNA library kit (Qiagen). Briefly, DNA was fragmented enzymatically into smaller fragments and desired insert size was achieved by adjusting fragmentation conditions. Fragmented DNA was end repaired and ‘A’s’ were added to the 3′ ends to stage inserts for ligation. During ligation step, Illumina compatible Unique Dual Index (UDI) adapters were added to the inserts and prepared library was PCR amplified. Amplified libraries were cleaned up, and QC was performed using Tapestation 4200 (Agilent Technologies). Libraries were sequenced on an Illumina NextSeq 500 or MiSeq platform to generate 2 × 150 or 2 × 250 bp reads respectively. Illumina reads were assembled into contigs using SPAdes^63^ and genes were called and annotated using Prokka (v1.14.6).^85^

Samples for Nanopore and Illumina hybrid assemblies were extracted using the NEB Monarch Genomic DNA Purification Kit. DNA was QC’ed using genomic Tapestation 4200. Nanopore libraries were prepared using the Ligation Sequencing Kit (SQK-LSK109), the Native Barcoding Expansions 1–12 (EXP-NBD104) and 13–24 (EXP-NBD114), and the NebNext Companion Module for Oxford Nanopore Technologies (E7180S). The shearing steps and first ethanol wash were eliminated to ensure high concentrations of long fragments. Using R9.4.1 flow cells, libraries were run on a MinION for 72 h at 180V. The Nanopore and Illumina hybrid assemblies were completed using Unicycler (v0.4.8)^64^ either with the untrimmed or trimmed Illumina reads. The assemblies with less number of circularized contigs were used for genome analysis.

#### Binary toxin genes prevalence analysis

*C. difficile* isolates (N = 827) from BioProject: PRJEB4556 were downloaded from NCBI, and assembled into contigs using SPAdes.^63^ A collection of 2143 *C difficile* genomes from Patric (date: Feb. 10 2021)^23^ were also downloaded. MLST was determined on those contigs by mlst.^86^ ST type with less than 3 isolates were removed. Binary toxin *cdtA*, *cdtB* and *cdtR* from R20291 (NCBI: NC_013316) were used as query to BLAST^65^ against the assembled contigs, and hits with at least 85% identity and 85% coverage of the query are considered a valid match.

#### UMAP (uniform manifold approximation and projection) analysis

A subset of isolate contigs of 199 ST1, 50 ST2, 50 ST3, 49 ST6, 50 ST8, 50 ST11, 42 ST14, 50 ST15, 50 ST17, 50 ST37 and 50 ST42, totaling 690 isolates were selected from the above Patric collection. They were all sequenced by short read technology, and they are the top 10 abundant ST groups except ST1 in the Patric collection. Genes were called and annotated from their contigs using Prokka (v1.14.6).^85^ By combining the 23 isolates from this study, we constructed a matrix of 731 isolates by 8025 annotated genes and hypothetical protein clusters. Specifically, hypothetical protein clusters were formed by clustering hypothetical proteins at 50% identity using cd-hit.^87,88^ Any protein sequences that were at least 50% similar fall into an artificially cluster. UMAP analysis was performed on the basis of the presence/absence of the genes/hypothetical protein clusters by setting the n_neighbors parameter to 675.

### QUANTIFICATION AND STATISTICAL ANALYSIS

Results represent means ± SD. Statistical significance was determined by t test and one-way ANOVA test. Multiple comparisons were corrected with False Discovery Rate with desired FDR at 0.05. Statistical analyses were performed using Prism GraphPad software v9.3.1 (* p < 0.05, ** p < 0.01, *** p < 0.001, **** p < 0.0001)

## Supplementary Material

1

2

3

## Figures and Tables

**KEY RESOURCES TABLE:** 

REAGENT or RESOURCE	SOURCE	IDENTIFIER

Bacterial and virus strains		

B. subtilis BS49	Wilson and Bott^58^	N/A
*C.difficile* R_cdtRKO10.3	This manuscript	BioSample: SAMN31149530
*C.difficile* R_cdtRKO8.1	This manuscript	BioSample:SAMN31149531
*C.difficile* R_cdtRmut6.1	This manuscript	BioSample:SAMN31149534
*C.difficile* R_cdtRmut8.1	This manuscript	BioSample:SAMN31149535
*C.difficile* R_cdtRstop4.2	This manuscript	BioSample:SAMN31149532
*C.difficile* R_cdtRstop8	This manuscript	BioSample:SAMN31149533
*C.difficile* R_phi75-2	This manuscript	BioSample:SAMN31149536
*C.difficile* R_phi75-2/3	This manuscript	BioSample:SAMN31149537
*C.difficile* R_phi75-3	This manuscript	BioSample:SAMN31149538
*C.difficile* R20291	Human isolate	NCBI:NC_013316
*C.difficile* ST1-10	Human isolate	BioSample:SAMN13566327
*C.difficile* ST1-11	Human isolate	BioSample:SAMN13566371
*C.difficile* ST1-12	Human isolate	BioSample:SAMN13566370
*C.difficile* ST1-19	Human isolate	BioSample:SAMN13566326
*C.difficile* ST1-20	Human isolate	BioSample:SAMN13566335
*C.difficile* ST1-23	Human isolate	BioSample:SAMN13566336
*C.difficile* ST1-25	Human isolate	BioSample:SAMN13566328
*C.difficile* ST1-26	Human isolate	BioSample:SAMN13566333
*C.difficile* ST1-27	Human isolate	BioSample:SAMN13566330
*C.difficile* ST1-35	Human isolate	BioSample:SAMN13566365
*C.difficile* ST1-49	Human isolate	BioSample:SAMN13566378
*C.difficile* ST1-5	Human isolate	BioSample:SAMN13566334
*C.difficile* ST1-53	Human isolate	BioSample:SAMN13566406
*C.difficile* ST1-57	Human isolate	BioSample:SAMN13566317
*C.difficile* ST1-58	Human isolate	BioSample:SAMN13566423
*C.difficile* ST1-6	Human isolate	BioSample:SAMN13566324
*C.difficile* ST1-62	Human isolate	BioSample:SAMN13566360
*C.difficile* ST1-63	Human isolate	BioSample:SAMN13566320
*C.difficile* ST1-65	Human isolate	BioSample:SAMN13566318
*C.difficile* ST1-67	Human isolate	BioSample:SAMN13566325
*C.difficile* ST1-68	Human isolate	BioSample:SAMN13566403
*C.difficile* ST1-69	Human isolate	BioSample:SAMN13566435
*C.difficile* ST1-75	Human isolate	BioSample:SAMN13566366
*C.difficile* VPI10463	ATCC	ATCC #43255
*E.coli* NEB5-alpha	NEB	NEB #C2987
*E.coli* NEBturbo	NEB	NEB #C2984
*E.coli* HB101(RP4)	Maikova et al.^59^	N/A

Chemicals, peptides, and recombinant proteins		

Metronidazole	Sigma-Aldrich	Cat# M3761
Neomycin Sulfate	Fisher scientific	Cat# BP2669
Vancomycin Hydrochloride	Hospira	UoS NDC # 00409-1319-01
Clindamycin hydrochloride	Sigma-Aldrich	Cat# C5296
Mitomycin C	Novus Biologicals	Cat# NB3258
Histodenz	Sigma-Aldrich	Cat# D2158
Amoxicillin/Potassium Clavulanate	NorthStar Rx	NDC# 16714029301
Chloramphenicol	Sigma-Aldrich	Cat# C1919
Thiamphenicol	Sigma-Aldrich	Cat# T0261
Taurocholic acid sodium salt hydrate	Sigma-Aldrich	Cat# T4009
Xylose	Sigma-Aldrich	Cat# X3877
Cefoxitin sodium salt	Sigma-Aldrich	Cat# C4786
D-Cycloserine	Sigma-Aldrich	Cat# C6880
L-Cysteine	Sigma-Aldrich	Cat# C7352
Minimum Essential Medium Eagle	Sigma-Aldrich	Cat# M8042
HEPES	Gibco	Cat# 845-1344
PEN/STREP	Gibco	Cat# 15140-122
L-Glutamine	Gibco	Cat# 810-1051
2-Mercaptoethanol	Applied Biosystems	Cat# AB1340
Gentamycin Sulfate	Gemini	Cat# 400-108

Critical commercial assays		

QuantiTect Reverse Transcription Kit	Qiagen	Cat# 205311
RNeasy PowerMicrobiome Kit	Qiagen	Cat# 26000
QiAamp PowerFecal pro DNA Kit	Qiagen	Cat# 51804
DNeasy PowerSoil Pro Kit	Qiagen	Cat# 47016
Gibson Assembly^®^ Cloning Kit	NEB	Cat# E5510S
QIAquick PCR Purification Kit	Qiagen	Cat# 28104
C. DIFFICILE TOXIN/ANTITOXIN KIT	TechLab	Cat# T5000
PowerTrack SYBR Green Master Mix	Thermo Fisher	Cat# A46109
QIAseq FX DNA library kit	Qiagen	Cat# 180479
NEB Monarch Genomic DNA Purification Kit	NEB	Cat# T3010S
Ligation Sequencing Kit	Oxford Nanopore	SQK-LSK109
Native Barcoding Expansions 1-12 and 13-24	Oxford Nanopore	EXP-NBD104 and EXP-NBD114
NebNext Companion Module for Oxford Nanopore Technologies	NEB	Cat# E7180S
Illumina MiSeq Reagent kit v2	Illumina	MS-102-2001

Deposited data		

Whole-genome sequencing assembly	This manuscript	BioProject: PRJNA885086 and PRJNA595724

Experimental models: Cell lines		

Hamster: CHO/dhFr-	ATCC	ATCC# CRL-9096

Experimental models: Organisms/strains		

Mouse: C57BL/6J	The Jackson Laboratory	JAX: 000664; RRID: IMSR_JAX: 000664
Mouse: inhouse Germ-free	University of Chicago	N/A
Mouse: MyD88~^/^~ (bred to C57BL/6J)	Adachi et al.^60^	N/A

Oligonucleotides		

See Table S1	IDT	N/A

Recombinant DNA		

Plasmid: pCE677	Kaus et al.^61^	N/A
Plasmid:pRPF144	Fagan and Fairweather^62^	Addgene# 106372
Plasmid:pRPF144-WTcdtR	This manuscript	N/A
Plasmid:pRPF144-MutcdtR	This manuscript	N/A

Software and algorithms		

SPAdes	Prjibelski et al.^63^	N/A
GraphPad Prism v. 9	GraphPad Software	N/A
Geneious Prime v.11	Geneious by Dotmatics	N/A
PATRIC web resources	Wattam et al.^23^	N/A
Unicycler v0.4.8	Wick et al.^64^	N/A
BLAST	Camacho et al.^65^	N/A
mlst	Jolley and Maiden^66^	N/A
FastANI (v 1.32)	Jain et al.^67^	N/A
seqtk	https://github.com/lh3/seqtk ^68^	N/A
snippy	Seemann. T, Snippy: rapid haploid variant calling, Githubhttps://github.com/tseemann/snippy ^69^	N/A
gubbins	Croucher et al.^70^	N/A
Anvi'o	Eren et al.^28^	N/A
PHASTER	Arndt et al.^71^	N/A
R	R Core Team (2022). R: A language and environment for statistical computing. R Foundation for Statistical Computing, Vienna, Austria. URLhttps://www.R-project.org/.	N/A
R package: tidyverse	Wickham et al.^72^	N/A
R package: Biostrings	Pages et al.^73^ R package version 2.64.1,<https://bioconductor.org/packages/Biostrings>.	N/A
R package: genoPlotR	Guy et al.^74^	N/A

## Highlights

- Clinical ST1 *C. difficile* isolates with identical PaLoc can differ remarkably in virulence
- Two avirulent *C. difficile* isolates remain avirulent in immune-deficient and germ-free mice
- Avirulent *C. difficile* isolates contain a deletion in the regulatory gene for binary toxin
- CdtR regulates Tcd and CDT toxins, thus broadly impacting the virulence of *C. difficile*
