# Supplementary material for: Virulence and genomic diversity among clinical isolates of ST1 (BI/NAP1/027) Clostridioides difficile
Source: Cell Rep. Author manuscript; Available in PMC 2023 Nov 6. (PMC10627504; doi:10.1016/j.celrep.2023.112861)
Supplement: 1 [file NIHMS1928240-supplement-1.pdf]

Cell Reports, Volume *42*

Supplemental information

Virulence and genomic diversity among clinical isolates of ST1 (BI/NAP1/027) *Clostridioides difficile*

Qiwen Dong, Huaiying Lin, Marie-Maude Allen, Julian R. Garneau, Jonathan K. Sia, Rita C. Smith, Fidel Haro, Tracy McMillen, Rosemary L. Pope, Carolyn Metcalfe, Victoria Burgo, Che Woodson, Nicholas Dylla, Claire Kohout, Anitha Sundararajan, Evan S. Snitkin, Vincent B. Young, Louis-Charles Fortier, Mini Kamboj, and Eric G. Pamer

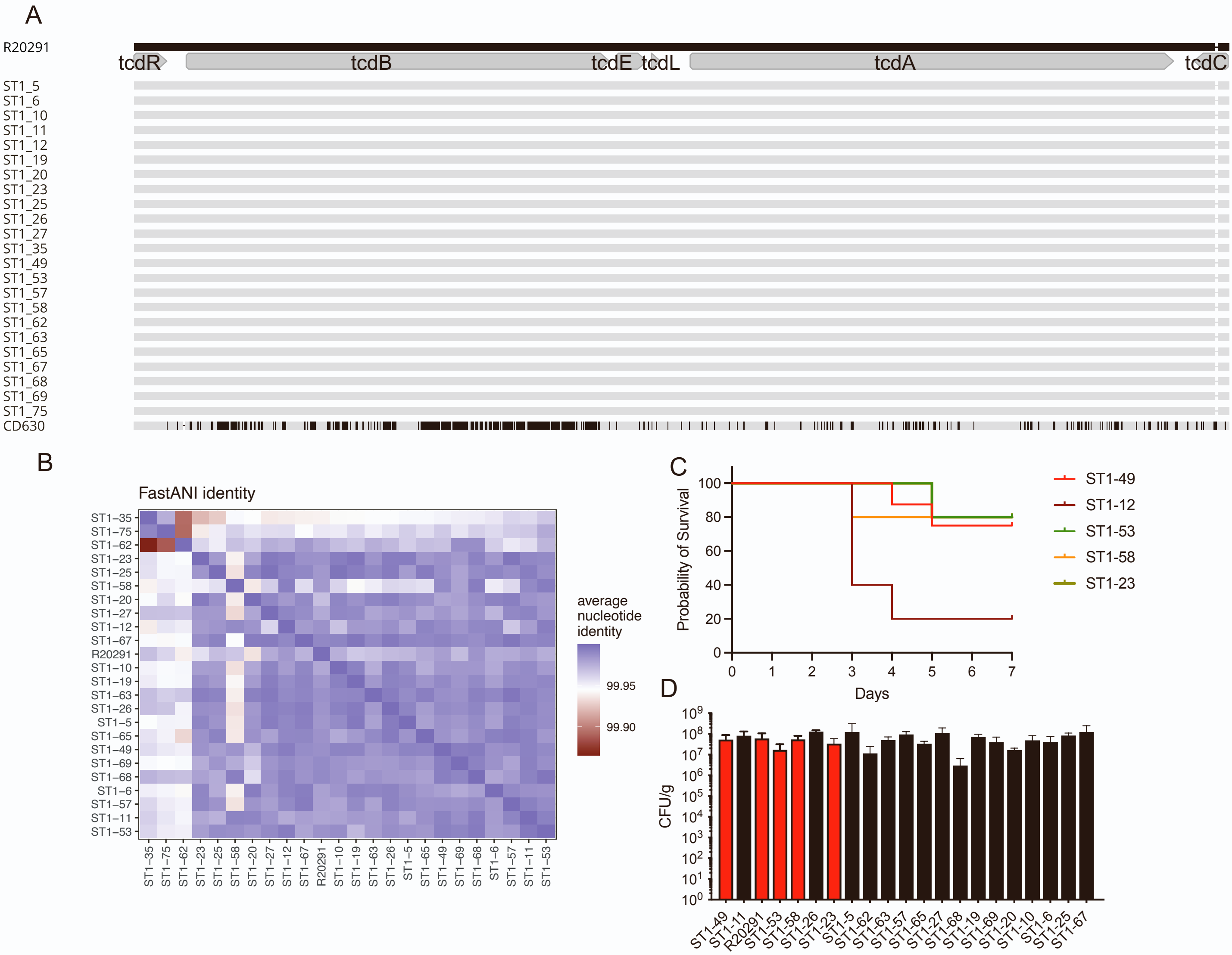

Figure S1. ST1 *C. difficile* strains are closely related. Relate to Figure 1. (A) Pathogenicity loci were extracted from whole-genome sequence and multiple sequence alignment was performed for all strains. Each dash indicates one single-nucleotide polymorphism. (B) Average nucleotide identity was calculated with pairs of ST1 isolates. (C) Survival curve of indicated strain over a 7-day time course post infection. (D) Fecal colony-forming units measured by plating on selective agar on 1 day post infection from Figure 1C. Results represent means ± SD.

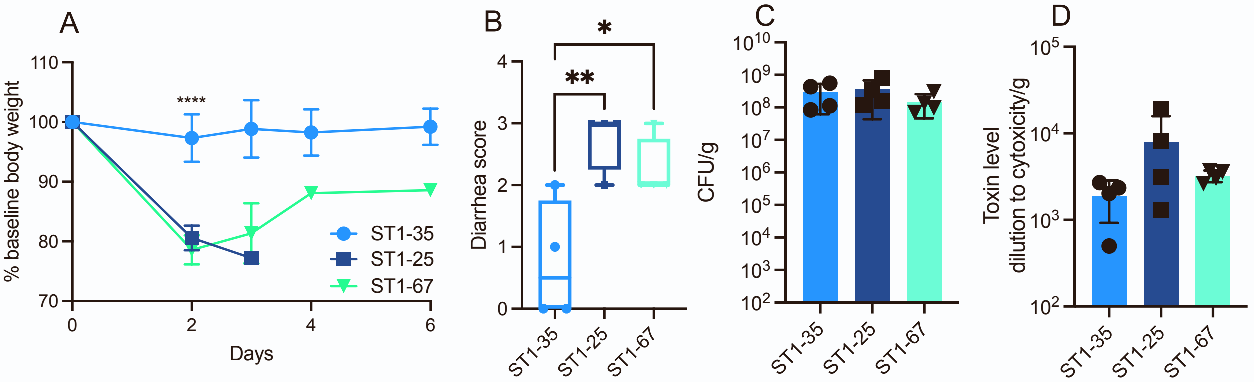

Figure S2. Avirulent *C. difficile* strain demonstrates no virulence in germ-free mice. Relate to Figure 3. Germ-free mice (n=4 per group) orally administered with 200 spores of indicated *C. difficile* strains. Daily body weight and acute disease scores were monitored for 6 days post infection. (A) %Weight loss to baseline of mice infected with indicated strains. (B) Diarrhea scores of mice infected with indicated strains on 3 days post infection. Results represent min to max showing all points. (C) Fecal colony-forming units measured by plating on selective agar 1 day post infection. (D) Fecal Tcd toxins measured by CHO cell rounding assay 1 day post infection. Results represent means ± SD. * $p < 0.05$, ** $p < 0.01$.

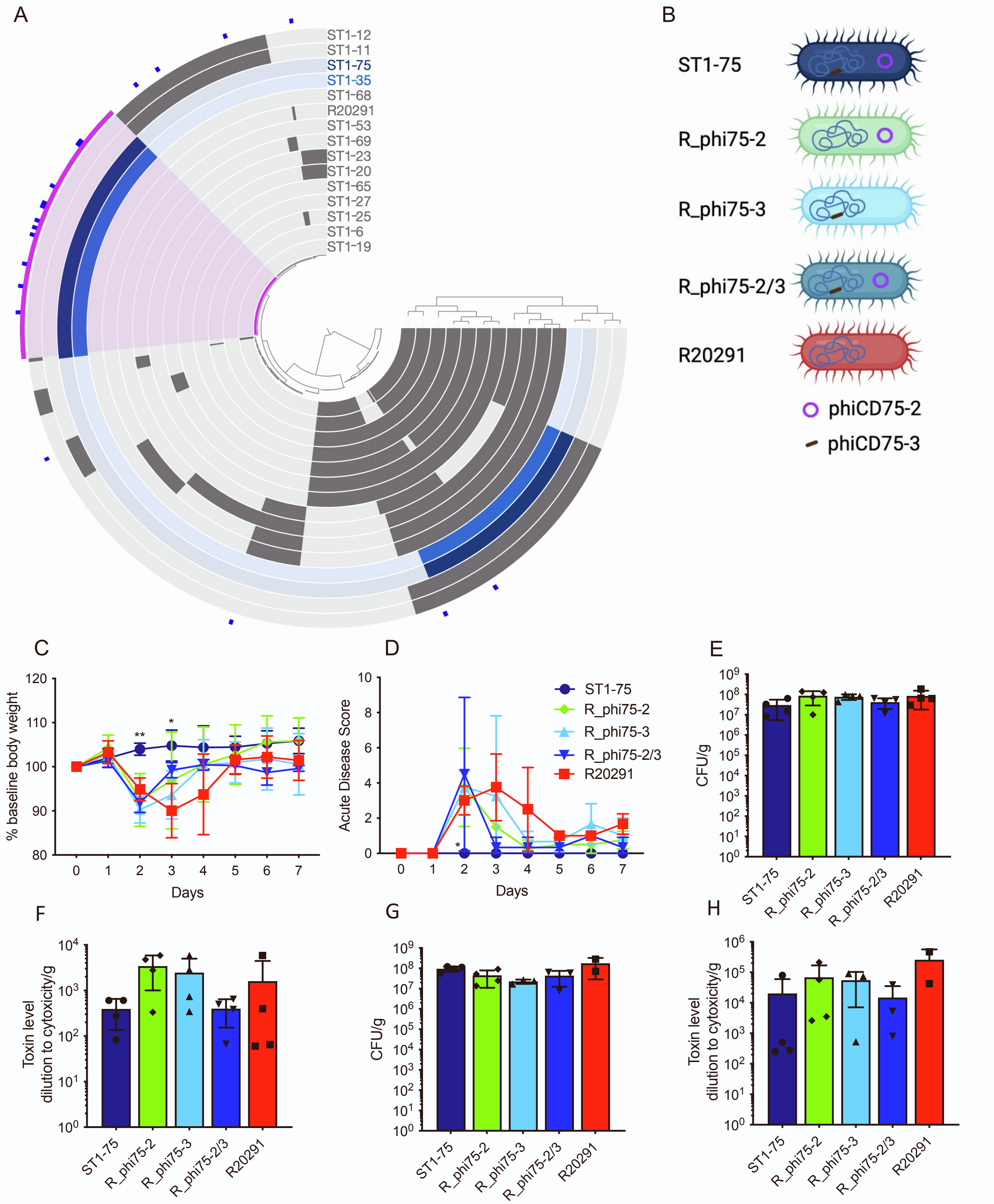

Figure S3. Prophages identified in avirulent *C. difficile* do not impact virulence in mice treated with antibiotics. (A) Anvi'o plot displaying accessory genomes of ST1 isolates. Highlighted gene clusters in purple are unique to ST1-35 and ST1-75. Blue dashes (outmost layer) indicate phage-related genes by NCBI COG. (B) Schematic of mutant strains made using R20291 *C. difficile* strain. (C-H) Wildtype C57BL/6 mice (n=4 per group) were treated with MNV and clindamycin as preciously described. Then, mice were inoculated with 200 *C. difficile* spores via oral gavage. Daily body weight and acute disease scores were monitored for 7 days post infection. (C) %Weight loss to baseline of mice infected with indicated strains. ST1-75 has significant difference on day 2 and day 3 compared to R20291. (D) Acute disease scores comprising weight loss, body temperature drop, diarrhea, morbidity of mice infected with indicated strains. ST1-75 has significant difference on day 2 compared to R20291. (E, G) Fecal colony-forming units measured by plating on selective agar 1 and 7 days post infection, respectively. (F, H) Fecal Tcd toxins measured by CHO cell rounding assay 1 and 7 days post infection, respectively. Results represent means ± SD. * $p < 0.05$, ** $p < 0.01$.

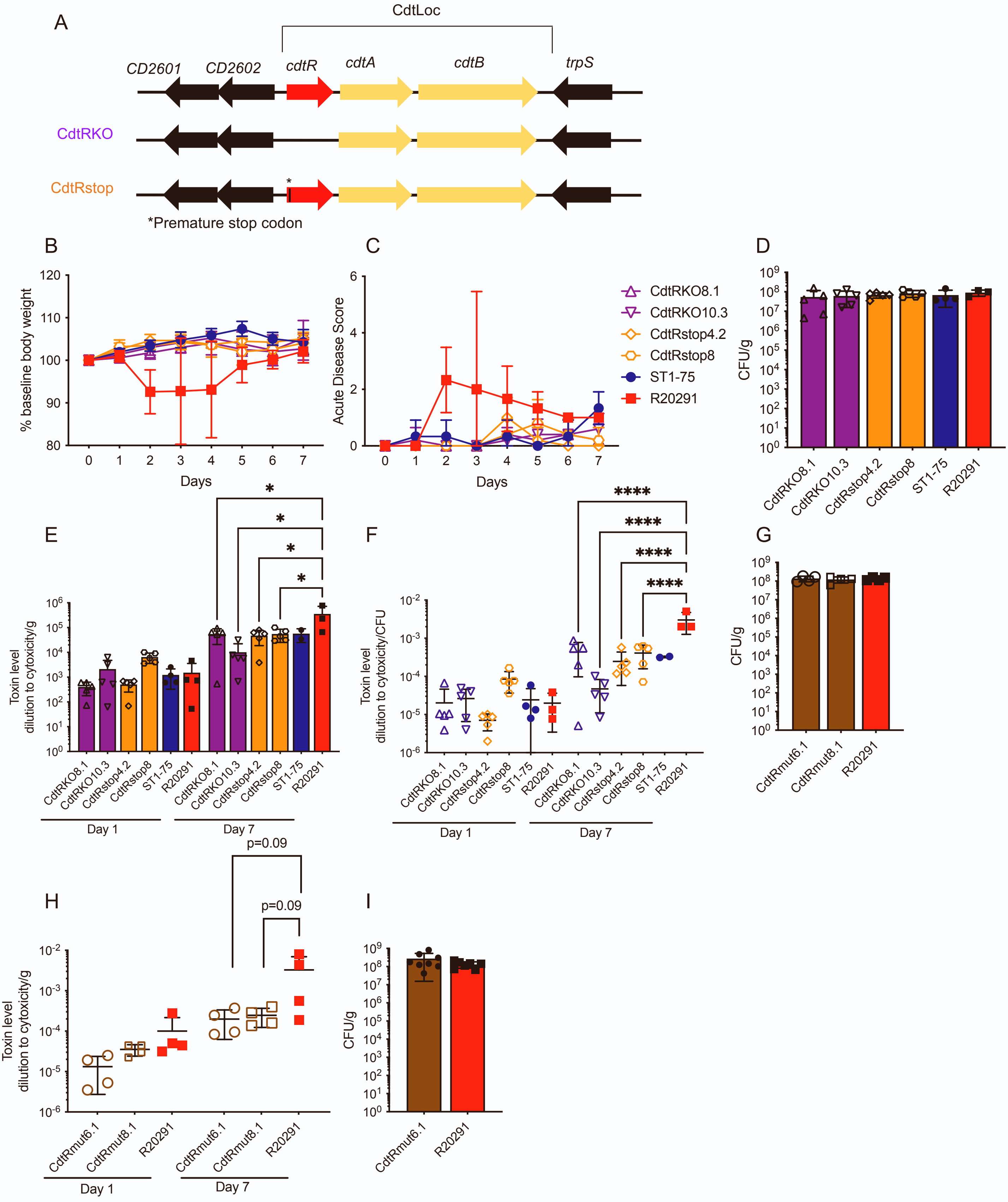

Figure S4. Binary toxin regulator *cdtR* does not impact *C. difficile* colonization in mice. Relate to Figure 4. (A) Schematic of *cdtR* mutants generated using R20291 *C. difficile* strain. (B-H) Wildtype C57BL/6 mice (n=3 to 5 per group) were treated with MNV and clindamycin as preciously described. Then, mice were inoculated with 200 *C. difficile* spores via oral gavage. Daily body weight and acute disease scores were monitored for 7 days post infection. (B) %Weight loss to baseline of mice infected with indicated strains. (C) Acute disease scores comprising weight loss, body temperature drop, diarrhea, morbidity of mice infected with indicated strains. (D, G) Fecal colony-forming units measured by plating on selective agar 1 day post infection (E) Fecal Tcd toxins measured by CHO cell rounding assay on indicated days. (F, H) Cecal Tcd toxin as in Figure S4E and 4E was normalized to CFU. (I) Germ-free mice (n=4) orally administered with 200 spores of indicated *C. difficile* strains. Fecal colony-forming units measured by plating on selective agar 1 day post infection. Results represent means ± SD. * $p < 0.05$, **** $p < 0.0001$.

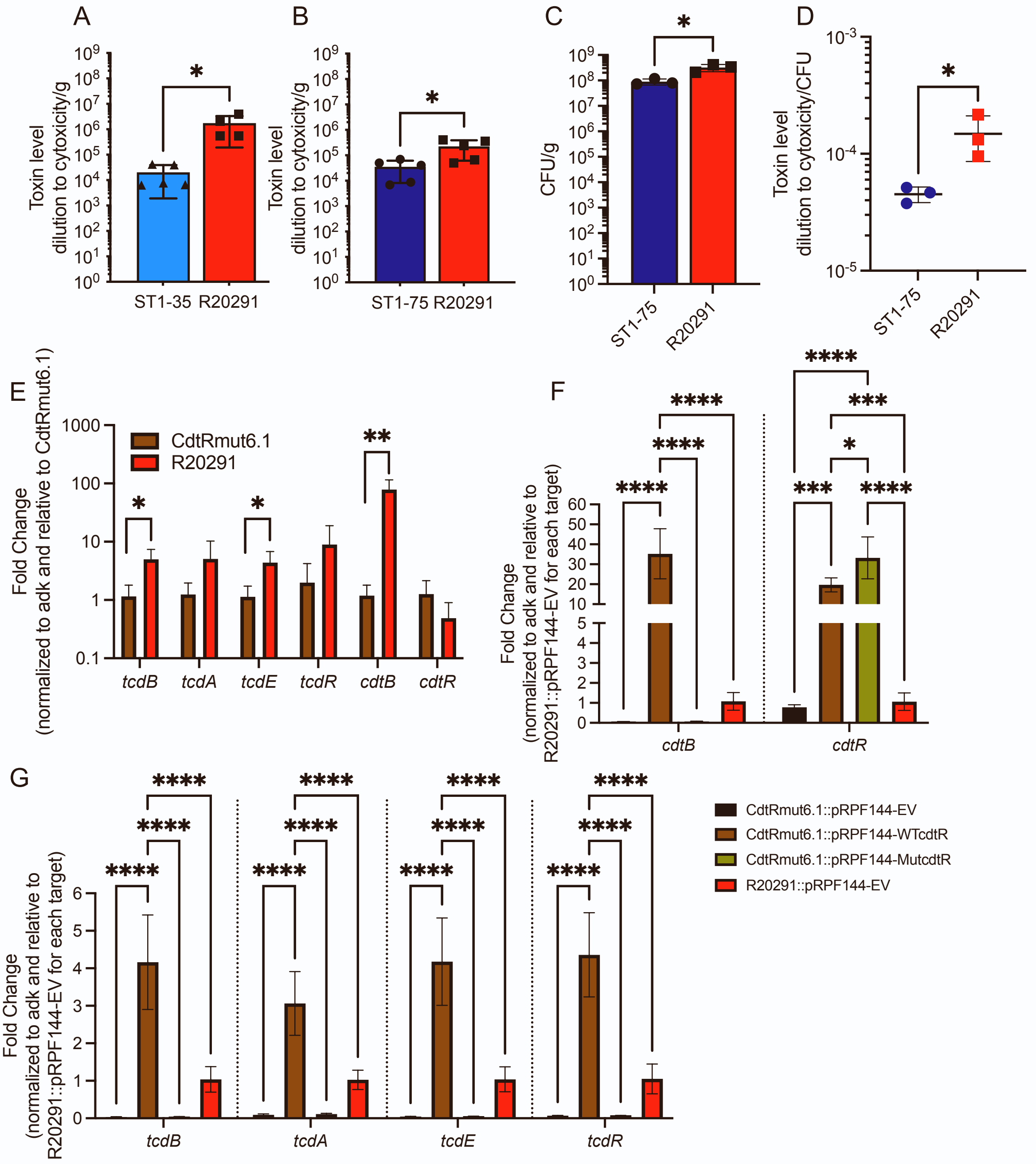

Figure S5. CdtR regulates PaLoc toxins production. Relate to Figure 5. (A-B) Wildtype C57BL/6 mice (n=3-5 per group) were treated with MNV and clindamycin as preciously described. Then, mice were inoculated with 200 *C. difficile* spores via oral gavage. (A) Fecal Tcd toxins measured by CHO cell rounding assay 7 days post infection for ST1-35. (B) Fecal Tcd toxins measured by CHO cell rounding assay 14 days post infection for ST1-75. (C-E) Germ-free mice (n=3-4) orally administered with 200 spores of indicated *C. difficile* strains and cecal contents were harvested at 24 hours post infection. (C) Cecal CFU measured by plating on selective agar. (D) Cecal Tcd toxin as in Figure 5A was normalized to CFU. (E) PaLoc and CdtLoc transcripts were measured by RT-qPCR with 2 technical replicates per target (except for tcdA with one technical replicate). Transcripts were all normalized to the *adk* and fold change is relative to CdtRmut6.1 for each of the genes. (F-G) PaLoc and CdtLoc transcripts were measured by RT-qPCR with 4 biological replicates. Transcripts were all normalized to the *adk* and fold change is relative to R20291::pRPF144-EV for each of the genes. Results represent means ± SD. * $p < 0.05$, ** $p < 0.01$, *** $p < 0.001$, **** $p < 0.0001$.

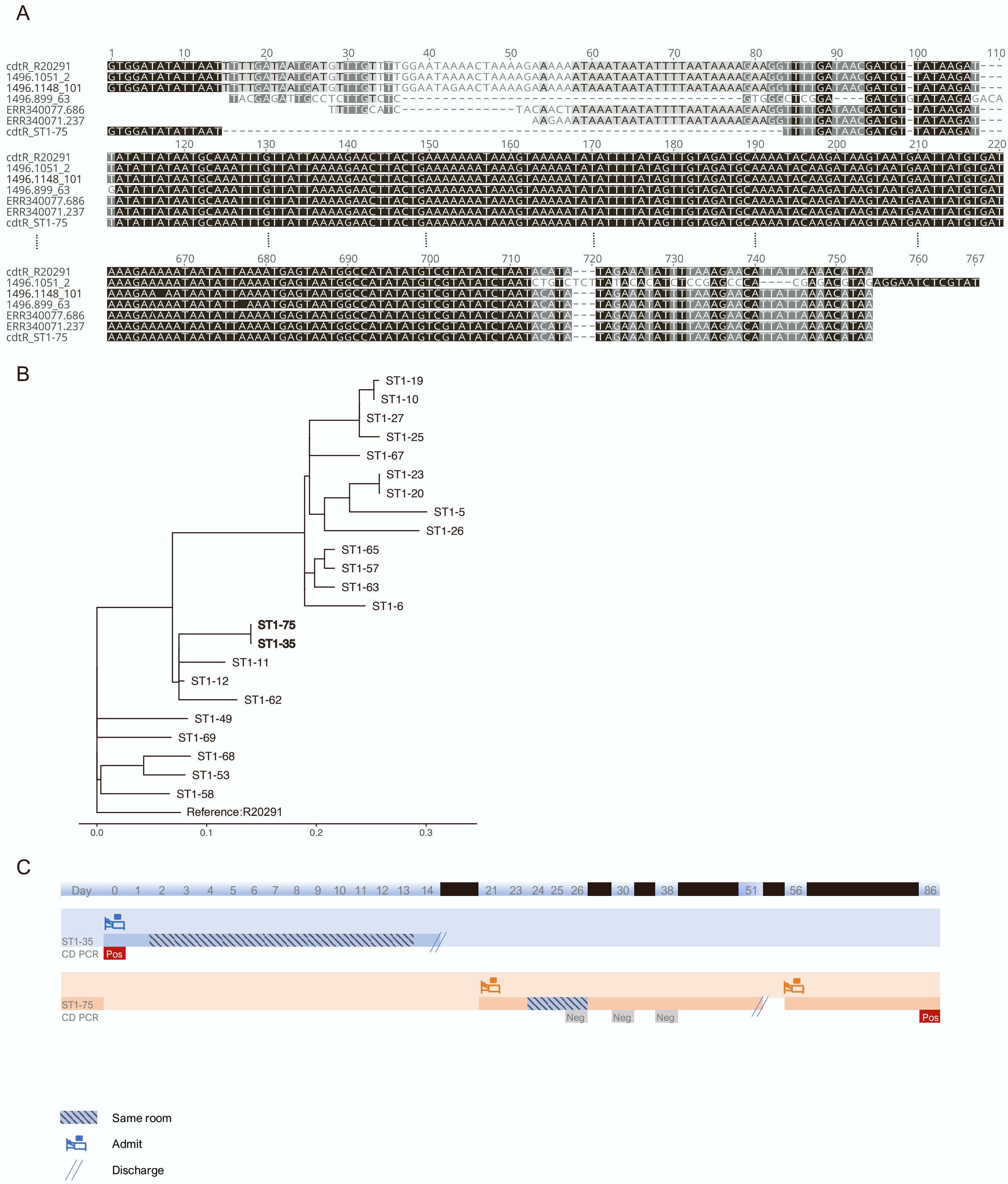

Figure S6. ST1-75/35 harbors unique mutations in *cdtR*. Relate to Figure 6. (A) *cdtR* hits without starting position at the beginning of *C. difficile* contigs were chosen to further examine their nucleotide differences in *cdtR* gene to R20291 and ST1-75. Five out of 491 ST1 isolates (from both strain collection databases) were found to have nucleotide variants. (B) Phylogenetic tree build based on core genome snps of ST1 isolates against R20291. (C) Timeline of hospital stay and spatial overlap between the two patients harboring ST1-75 and ST1-35.

Name	Sequence 5'-3'	Reference	Purpose
tcdA_qFor	GTATGGATAGGTGGAGAAGTCA	Babakhani et al. [81]	Forward primer for tcdA transcription analysis
tcdA_qRev	CTCTTCCTCTAGTAGCTGTAATGC	Babakhani et al. [81]	Reverse primer for tcdA transcription analysis
tcdB_qFor	AGCAGTTGAATATAGTGGTTTAGTTAGAGTTG	Wroblewski et al. [82]	Forward primer for tcdB transcription analysis
tcdB_qRev	CATGCTTTTTTAGTTTCTGGATTGAA	Wroblewski et al. [82]	Reverse primer for tcdB transcription analysis
tcdE_qFor	ATAAACCTAGGAGGCGTTATGAATATGA	Edwards et al. [79]	Forward primer for tcdE transcription analysis
tcdE_qRev	TTATTGCACTTAAACATCCTAATAATGTATCAAA	Edwards et al. [79]	Reverse primer for tcdE transcription analysis
tcdR_qFor	AGCAAGAAATAACTCAGTAGATGATT	Edwards et al. [79]	Forward primer for tcdR transcription analysis
tcdR_qRev	TTATTAAATCTGTTTCTCCCTCTTCA	Edwards et al. [79]	Reverse primer for tcdR transcription analysis
cdtB_qFor	GCAGTTAAGTGGGAAGATAG	Angione et al. [80]	Forward primer for cdtB transcription analysis
cdtB_qRev	TCCATACCTACTCCAACAAT	Angione et al. [80]	Reverse primer for cdtB transcription analysis
cdtR-2_qFor	TTGAAACAAGCGCTATTCCACA	This study	Forward primer for cdtR transcription analysis
cdtR-2_qRev	TGTACACGAATAAAGCATGCATC	This study	Reverse primer for cdtR transcription analysis
rpsJ_qFor	GATCACAAGTTTCAGGACCTG	Metcalf et al. [83]	Forward primer for rspJ transcription analysis
rpsJ_qRev	GTCTTAGGTGTTGGATTAGC	Metcalf et al. [83]	Reverse primer for rspJ transcription analysis
adk_qFor	GTGTATGTGATGTATGCCAAG	Metcalf et al. [83]	Forward primer for adk transcription analysis
adk_qRev	CCTAAGGCTGCGACAATATC	Metcalf et al. [83]	Reverse primer for adk transcription analysis
R_cdtR_up_For	AAACAGCTATGACCGCGGCCGCCTAAACACACATTATC ATCTCTCTG	This study	Forward primer amplifying upstream region of cdtR gene for cdtRKO, cdtRstop and cdtRmut
R_cdtRKO_up_Rev	AACTTTCAGTTTAGCGGTCTGGGCGCCTAAATACCCTCC TATAAAAAAATTCAAAAG	This study	Reverse primer amplifying upstream region of cdtR gene for cdtRKO
R_cdtRKO_down_For	GGCGCCCAGACCGCTAAACTGAAAGTTTAAATAGAAAA AAGAGATGTCTCAAGATAAG	This study	Forward primer amplifying downstream region of cdtR gene for cdtRKO
R_cdtRKO_down_Rev	TTATTTTTATGCTAGCTCGAGTAAGTCTTGTGCATAAAT GTTATTAGG	This study	Reverse primer amplifying downstream region of cdtR gene for cdtRKO
R_cdtRstop_up_Rev	AACTTTCAGTTTAGCGGTCTGGGCGCCTTATCAAAAATT AATATATCCACTAAATACCC	This study	Reverse primer amplifying upstream region of cdtR gene for cdtRstop
R_cdtRstop_down_For	GGCGCCCAGACCGCTAAACTGAAAGTTTTTTGATAACG ATGTTATAAGATTATATTATAA	This study	Forward primer amplifying downstream region of cdtR gene for cdtRstop
R_cdtRstop/mut_down_Rev	TTATTTTTATGCTAGCTCGAGATCTGATAAAGACCTTAA ACTTTTATAG	This study	Reverse primer amplifying downstream region of cdtR gene for cdtRstop and cdtRmut
R_cdtRmut_up_Rev	TTATAATATAATCTTATAACATCGTTATCAAAAATTAATA TATCCACTAAATACCC	This study	Reverse primer amplifying upstream region of cdtR gene for cdtRmut
R_cdtRmut_down_For	GGGTATTTAGTGGATATATTAATTTTTGATAACGATGTT ATAAGATTATATTATAA	This study	Forward primer amplifying downstream region of cdtR gene for cdtRmut
R_cdtR_sgRNA1c_For	AATTAAACTGTAAATGGCCAAATAATATTTTAATAAAAG AGTTTTAGAGCTAGAAATAGC	This study	Forward primer cloning gRNA into pCE677 digested with MscI and MluI
CDEP3876	AACCATCTAAAAATAGTTGCAGAGCTTACGCGTC	Kaus et al. [61]	Universal Reverse primer for gRNA Cloning into pCE677 digested with MscI and MluI
LCF0312	AGCGGTATCGGCTTGGTTGTAGAT	This study	Verify phage identity for phiCD75-2
LCF0313	TGCTAGTTTCCTGTCAAGGTCGCT	This study	Verify phage identity for phiCD75-2
LCF1242	CGACCCACCTAAAGGTATTCA	This study	Verify phage identity for phiCD75-3
LCF1243	GTTCTTTAGTCCAGTTCCCATTTC	This study	Verify phage identity for phiCD75-3
pRPF144cdtR_For	GAATTCTGCATCAAGCTAGCTTGGTACCAATTAGAAGTT AAATAATTC	This study	Forward primer cloning CdtR into pRPF144 digested with KpnI and BamHI
pRPF144cdtR_Rev	CACACTGGCGGCCGTTACTAGTGGATCCTTATGTTTTAA TAATGTTC	This study	Reverse primer cloning CdtR into pRPF144 digested with KpnI and BamHI

Table S1 Oligonucleotides used in this study
